# A Functional Perspective Analysis of Macroalgae and Epiphytic Bacterial Community Interaction

**DOI:** 10.3389/fmicb.2017.02561

**Published:** 2017-12-22

**Authors:** July Z. Florez, Carolina Camus, Martha B. Hengst, Alejandro H. Buschmann

**Affiliations:** ^1^Programa de Doctorado en Ciencias mención Conservación y Manejo de Recursos Naturales, Universidad de Los Lagos, Puerto Montt, Chile; ^2^Centro i~mar and CeBiB, Universidad de Los Lagos, Puerto Montt, Chile; ^3^Departamento de Ciencias Farmacéuticas, Universidad Católica del Norte and CeBiB, Antofagasta, Chile

**Keywords:** epiphytic bacteria, macroalgae, holobiont, biological interactions, host-specific

## Abstract

Macroalgae are photosynthetic, multicellular, sessile eukaryotic organisms that offer diverse habitats for the colonization of epiphytic bacteria, therefore establishing biological interactions of diverse complexity. This review focusses on the interactions between macroalgae and their Epiphytic Bacterial Community (EBC); the main aims are to ascertain whether (1) the epiphytic bacterial groups differ at the phylum and genus levels of the macroalgae; (2) the methodologies used so far to study these microorganisms are related in any way to eventual variations of the EBCs on macroalgae; and (3) the EBC of macroalgae has a functional means rather a simple taxonomic grouping. Results showed firstly the taxonomic grouping of macroalgae does not explain the composition and structure of the EBCs. Secondly, the methodology used is important for describing EBCs; and thirdly, multiple bacteria can have the same function and thus to describe the functionality of EBCs it is important to recognize host-specific and generalist bacteria. We recommend the incorporation of a complementary approach between the taxonomic composition and the functional composition analyzes of EBCs, as well as the use of methodological tools that allow analysis of interactions between the EBCs and their hosts, based on the “holobiont” concept.

## Introduction

Macroalgae are photosynthetic, multicellular, sessile eukaryotic organisms that play an important role in marine ecosystems as primary producers and habitat engineers, providing shelter and food for various organisms (i.e., Bulleri et al., 2002; Fraschetti et al., 2006; Almanza et al., 2012). Macroalgae surface represents a suitable biotic habitat, being constantly colonized by microscopic stages of different types of epibionts (i.e., Buschmann et al., 1997; Rao et al., 2007) and microorganisms (Bolinches et al., 1988; Jensen et al., 1996). Different associations between bacteria and macroalgae have been the subject of research during past decades, such as studies undertaken by Provasoli and Pintner (1980) showing that the growth of bacteria with morphogenic activity depends on the production of exudates by macroalgae, implying that some metabolites released by the alga may be precursors or activators of adaptive enzymes producing morphogenetic substances. One of the principal interactions documented in the literature, reveals defense mechanisms of macroalgae controlling the formation of bacterial biofilms on their surfaces (Lu et al., 2008; Nylund et al., 2008). Nevertheless, the importance of taking a functional focus on the complex ecological interactions that these microorganisms can establish with their hosts is only recently emerging (Zhang et al., 2015).

Bearing in mind the enormous diversity of bacteria that inhabit marine ecosystems (Whitman et al., 1998), and the heterogeneity of habitats provide by macroalgae as substrate (Wahl et al., 2012), opportunities to establish symbiotic relationships between these two groups are particularly frequent (Grossart, 2010; Wichard, 2015). This is supported by several studies showing that the composition and structure of macroalgae surface-associated bacterial communities differ from those found in the surrounding water column (Morán et al., 2008; Staufenberger et al., 2008; Lachnit et al., 2009a; Bengtsson et al., 2010; Michelou et al., 2013; Mancuso et al., 2016). Similarly, the specificity of microorganisms associated with different macroalgae groups have also been reported. Lachnit et al. (2009a) studied the epiphytic bacterial communities of the species *Delesseria sanguinea, Fucus vesiculosus, Saccharina latissima (formerly Laminaria saccharina)* and *Ulva compressa* in two different habitats, and reported greater similarity between bacterial communities of the same macroalgal species inhabiting different environments, compared to those sharing the same habitat. Similar results were described by Hengst et al. (2010) for *U. compressa* and *Lessonia nigrescens*. Also, Barott et al. (2011) showed that macroalgal genus belonging to different phyla were colonized by specific bacterial communities. According to the literature, the different physiological and biochemical properties of different macroalgal groups could provide some explanation for the specificity of bacteria-macroalgae interactions (Beleneva and Zhukova, 2006; Goecke et al., 2010). For example, secondary metabolites found in macroalgae may be associated both with defense mechanisms against some bacterial groups (Armstrong et al., 2001; Lam et al., 2008; Wiese et al., 2009), and facilitation mechanisms, favoring the settlement of other microorganisms (Lachnit et al., 2009b). In addition to these interactions, epiphytic bacteria fulfill a wide variety of functions for their host, such as: providing growth factors and vitamins (Provasoli and Pintner, 1980; Croft et al., 2005; Singh et al., 2011), nitrogen fixation (Penhale and Capone, 1981), and pathogenic activity (Vairappan et al., 2001) among others. In view of the aforementioned, it would appear that the ecology and development of macroalgae cannot be fully understood without considering the interaction with their associated microorganisms (Egan et al., 2013). For this reason, various authors have suggested the use of the holobiont concept as a perspective to analyze and understand the result of these interactions (Barott et al., 2011; Egan et al., 2013; Aires et al., 2015).

In this context, the methodology used to study the EBCs associated to macroalgae plays a very important role, since the resolution obtained by the methodology selected may leave out relevant groups of microorganisms. Traditionally, culture-dependent and microscopy methods have been used to obtain information about the composition and structure of these communities (Friedrich, 2012). Nevertheless, it has been shown that only <1% of the bacteria found in the natural environment are cultivable (Eilers et al., 2000). From the nineties onwards, molecular tools have been incorporated based on the polymorphism of the 16S rRNA gene, such as FISH (Tujula et al., 2010), DGGE (Longford et al., 2007; Bengtsson et al., 2010), and clonal analysis and Sanger sequencing (Fisher et al., 1998; Ashen and Goff, 2000; Meusnier et al., 2001; Hengst et al., 2010) to characterize the epiphytic microorganisms of macroalgae. These methods are a higher degree of more sensitivity compared to previous ones, allowing a more precise identification of the different taxa. The need to identify the function carried out by these bacteria, both in the environment and their hosts, is currently being analyzed, thanks to the recent incorporation of molecular methodologies with even higher resolution, such as deep sequencing techniques (e.g., Pyrosequencing and Illumina; Luo et al., 2012). These techniques, make possible to obtain more specific and objective information to describe the bacterial composition of natural environments, using community DNA samples obtained directly from macroalgal surfaces. In addition, the study of these communities from a functional perspective, have glimpsed the effect of these microorganisms on their hosts and, in turn, identified possible responses of the host to these effects (Rosenberg et al., 2010; Burke et al., 2011a).

The previously described aspects highlight the importance of addressing issues regarding the specificity of the bacterial groups associated with the macroalgae, the levels of taxonomic resolution used to analyze possible biological interactions and the incorporation of functional information in explanations relating the composition and structure of epiphytic bacterial communities. For these reasons, the present review focusses on establishing the state of the art regarding the knowledge of the interactions between macroalgae and their epiphytic bacterial communities. The main objectives of this review are determining whether (1) the epiphytic bacterial groups differ at the phylum and family level and according to the macroalgae genus; (2) the methodologies used for the study of these microorganisms are related to the possible differences between the different groups of macroalgae; and (3) whether the epiphytic bacterial groups associated with the macroalgae functionally interact beyond a purely taxonomic approach.

## Materials and methods

The information analyzed in this study was based on research articles obtained through the web search engine of the Google Scholar database, using the following keywords: “seaweed” OR “macroalgae” OR “epiphytic” OR “bacterial” OR “communities” and the total coincidence “epiphytic bacterial communities on seaweed,” from which the oldest article found was from 1970. The selection of these keywords was based on the objective of this work oriented to the search of literature in which the epiphytic bacteria were considered from a community approach and not as isolated strains. The documents were filtered using the following selection criteria: (1) minimum information such as the identification of the bacterial groups at phylum level, (2) well-described methodology used to detect the bacterial groups. A total of 72 studies were obtained by this search and only 32 fulfilled the above described criteria.

The studies selected were categorized based on (1) macroalgal phylum (Chlorophyta, Heterokontophyta, and Rhodophyta); (2) macroalgal genus; and (3) methodology used [culture-dependent methods (CDM), molecular methods (MM), culture-dependent + molecular methods (CDM + MM), Pyrosequencing, and Illumina-Mi-Seq]. The analysis of the traditional molecular methods and the Next Generation Sequencing (NGS) technologies (Pyrosequencing and Illumina) was carried out separately due to the differences in the detection of bacterial taxa that are found in low abundances within the communities. The information related to the bacterial groups was treated as presence/absence data in each article consulted, both at the phylum and family level. In addition, classes of proteobacteria were included in the analysis. The classification of bacterial taxa identified for each macroalgae were considered as described by each author in the original paper. The percentages of identity 84, 86, and 92% were considered for the taxonomic assignment at phylum, class and family level, respectively, as suggested by Yarza et al. (2014).

Different approaches were used to analyze the information. To compare the Epiphytic Bacterial Community (EBC) at the phylum and class level, a Venn diagram was created, using Venny 2.1 online software (Oliveros, 2007), which provided the percentage of bacterial taxa (phylum and Proteobacteria class) present in each group of macroalgae, calculated based on the number of total taxa found in the literature consulted. A comparative analysis of the EBCs among different macroalgal genus was undertaken using heatmap-type graphs. For this purpose, abundance was defined as the frequency of appearance of different epiphytic bacterial taxa reported for each macroalgal genus in the literature consulted. In addition, the following scale was defined for the analysis of the information: rare bacteria were those mentioned only once in the literature and common bacteria are those that appear at least mentioned twice in the literature. Finally, a cluster analysis was used to group the data, based on a Bray-Curtis dissimilarity matrix, using the average distance criterion (http://cc.oulu.fi/~jarioksa/opetus/metodi/sessio3.pdf) and a cut off was assigned on each case, based on the clustering obtained for the different categories of the specific analysis (i.e., macroalgae phylum, used methodology). The latter was undertaken using R v0.99.892 statistical software (http://www.R-project.org). Information for analysis of epiphytic bacteria functionality was only obtained from 23 of the studies identified, which focused on evaluating some type of specific function as specified in the methodology of each of the selected papers.

## Results

Between 1970 and 2016, 32 studies related to epiphytic bacteria on macroalgae have been published incorporating the key words and the selection criteria used in this review. The investigations described a total of 24 macroalgal genera associated with epiphytic bacteria, 13 Heterokontophyta (54.2%), 8 Rhodophyta (33.3%), and 3 Chlorophyta (12.5%; Table S2).

**Figure 1:**
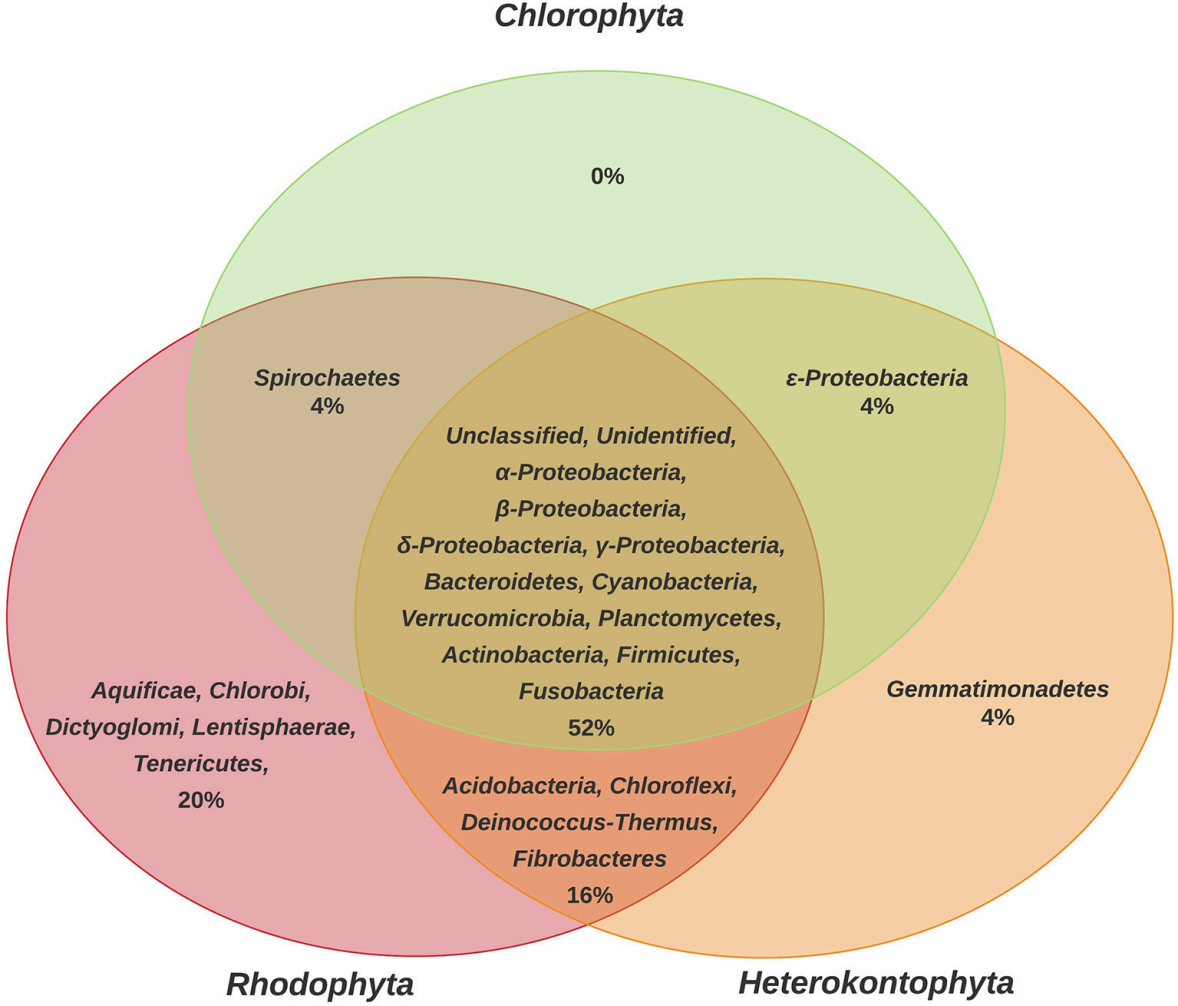
Comparison of phyla of epiphytic bacteria reported for the different phyla of macroalgae (Chlorophyta, Heterokontophyta, and Rhodophyta). Proteobacteria classes were included because some articles included this taxonomic level. The percentage values were calculated based on the total number of bacterial phyla found in the literature consulted (*n* = 25) and represented in a Venn Diagram to indicate the bacteria that are shared among macroalgal phyla, as well as the methodology used in each of the 25 studies (Table S1).

The results obtained show that Heterokontophyta, Chlorophyta, and Rhodophyta macroalgae, shared 52% of the bacteria (Figure 1). Some bacterial phyla were only reported in certain macroalgal groups, such as Aquificae, Chlorobi, Dyctioglomi, Lentisphaerae, and Tenericutes for Rhodophyta (20%) and Gemmatimonadetes for Heterokontophyta (4%).

Analyzing the information at macroalgal genus level and considering the bacterial taxonomic assignment at the phylum and class level, the *Laurencia* (Rhodophyta) genus presented the greatest bacterial diversity (21 taxa), followed by *Ulva* (Chlorophyta) (16 taxa) and *Laminaria* (Heterokontophyta) (13 taxa). While some genera with less diversity correspond to *Tauya, Alaria, Arthrothamnus, Desmarestia, Splachnidium*, and *Chordaria* (Heterokontophyta) and *Asparagopsis, Polysiphonia*, and *Camphylaephora* (Rhodophyta).

**Figure 2:**
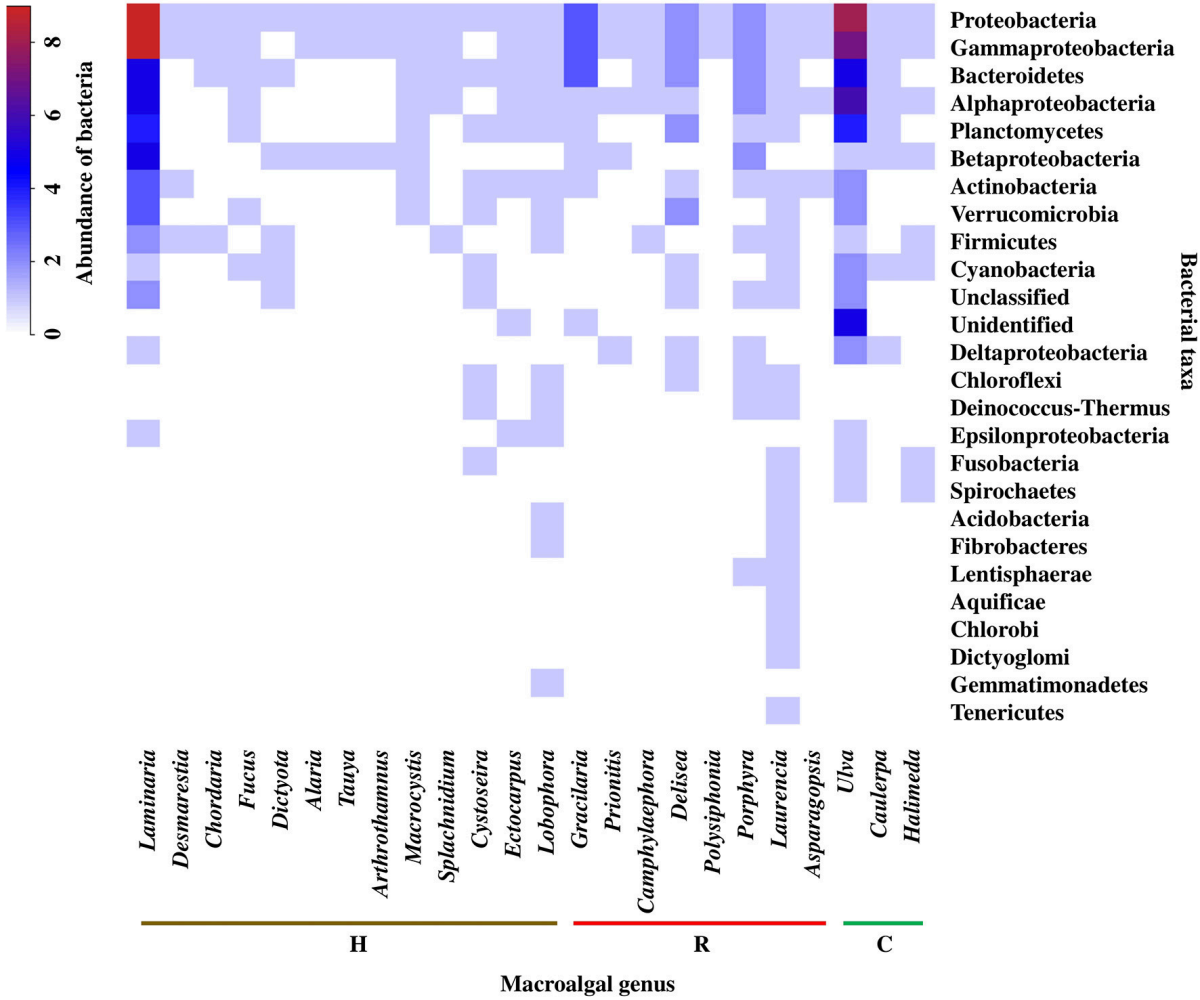
Abundance and distribution of epiphytic bacteria, at the level of phylum and class of Proteobacteria, associated to macroalgal genera of the different macroalgal phyla: Chlorophyta (C), Heterokontophyta (H), and Rhodophyta (R). Proteobacteria classes were included because some articles included this taxonomic level. The scale in the upper left shows the correspondence between colors and abundance values of bacteria. Abundance was defined as the number of Presences/Absences of epiphytic bacteria reported for each macroalgal genus in the literature consulted (*n* = 32). The bacterial phyla were ordered from highest to lowest abundance value. “Unclassified” are microorganisms that could not be classified in any group. “Unidentified” classified as bacteria, but could not be strongly identified. Taxonomic classification corresponded to those used in the literature consulted (Tables S2, S3).

Regarding the distribution patterns of bacterial groups in the different macroalgal genera, it was observed that Proteobacteria was the only phylum present in all macroalgal genera. However, within the Proteobacteria, the classes Alphaproteobacteria, Betaproteobacteria, Deltaproteobacteria, Gammaproteobacteria, and Epsilonproteobacteria showed variations, both within and among different macroalgal genera. According to the above, the highest bacterial abundances were found in the genus *Laminaria* and *Ulva*, corresponding to the Gammaproteobacteria and Alphaproteobacteria, as well as the Bacteroidetes and Planctomycetes. In general, the highest values for bacterial abundance were associated with genera of different macroalgal phyla, *Laminaria* and *Delisea* (Heterokontophyta), *Gracilaria* and *Porphyra (*currently *Pyropia)* (Rhodophyta), and *Ulva* (Chlorophyta) (Figure 2). In addition, the formation of 3 groups was observed (cut off point = 45% similarity) (Figure S1), but there was no discrimination among macroalgal phyla (Figure 2). For example, a higher similarity was found between the genera *Laminaria* and *Ulva* (different phyla) than *Laminaria* and *Macrocysti*s or between *Ulva* and *Halimeda*, which belong to the same phyla (Figure 2).

**Figure 3:**
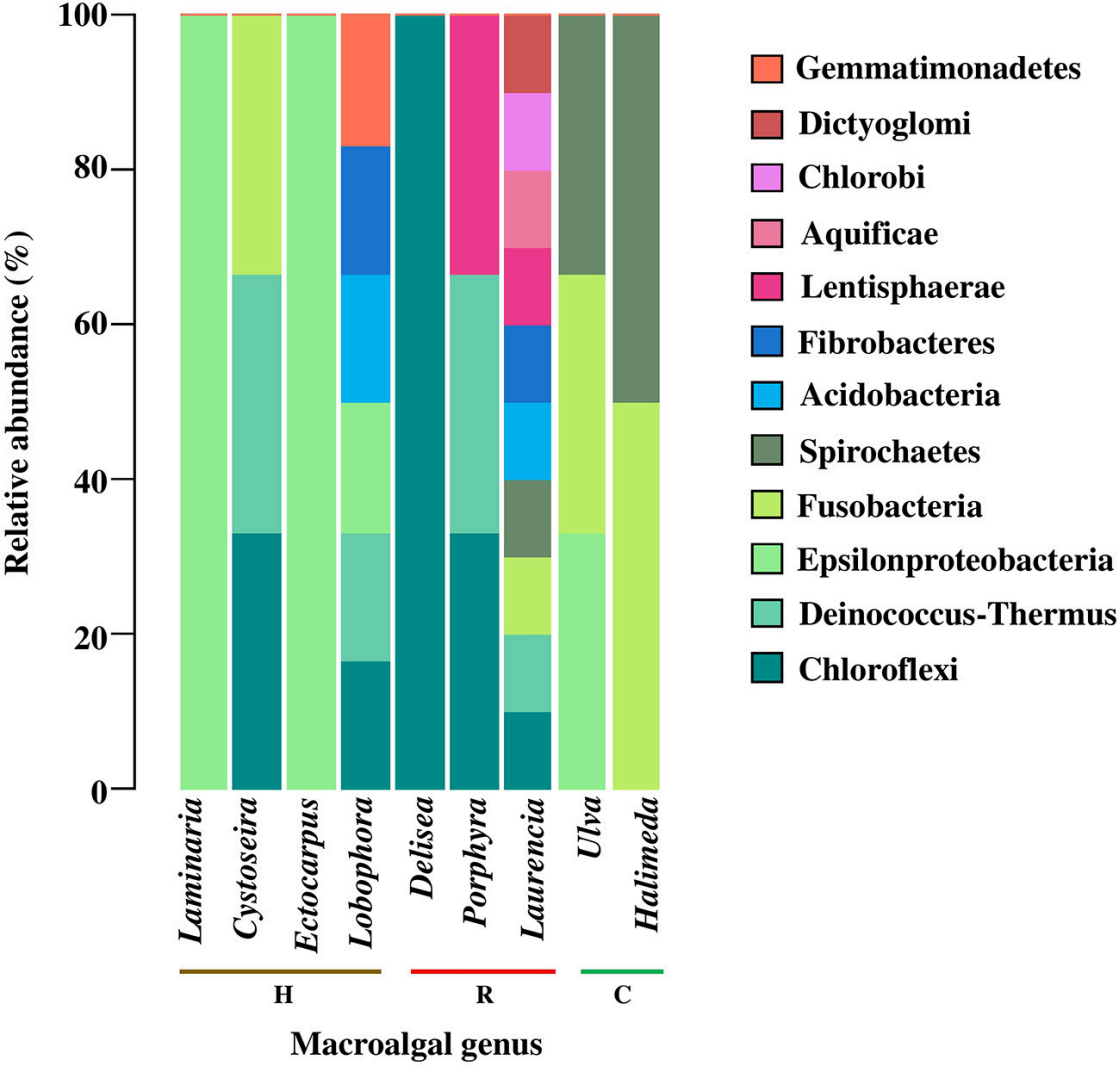
Relative abundance of rare epiphytic bacteria, at the phylum level and class of Proteobacteria, associated to macroalgal genera of the different macroalgal phyla: Chlorophyta (C), Heterokontophyta (H), and Rhodophyta (R). Proteobacteria classes were included because some articles included this taxonomic level. Rare epiphytic bacteria were defined as those that were only mentioned once in the literature consulted. The percentage values for each bacterial phylum were calculated in relation to the total number of rare epiphytic bacteria associated with each macroalgal genus according to the literature consulted (*n* = 32). Bacterial phyla were ordered from more to less common among macroalgal genus. The taxonomic classification corresponded to those used in the literature consulted (Table S4).

Bearing in mind that many bacteria phyla and classes are shared among different macroalgal genera, (value = 1, color key of Figure 2), we present a specific description for them in Figure 2. Rare bacterial taxa (i.e., those reported only once) were present in only 9 of the 24 macroalgal genera analyzed, corresponding to 4 for Heterokontophyta, 3 for Rhodophyta, and 2 for Chlorophyta (Figure 3). The genera *Laurencia* and *Lobophora* present a higher number of rare bacterial taxa than the rest of the macroalgae. Some of these were exclusively described for genus *Laurencia*, for example Aquificae, Chlorobi, and Dictyoglomi, and for genus *Lobophora* the group Gemmatimonadetes. While in the genera *Cystoseira, Porphyra, Ulva, Halimeda*, and *Laminaria*, at least 2–3 bacterial taxa were observed for each macroalgal genus. On the contrary, in other macroalgae, such as *Delisea* and *Ectocarpus* there is only a few rare bacteria, represented by the Cloroflexi and Epsilonproteobacteria groups, respectively. It is worth to mention that more studies have been carried out on some particular macroalgal genera, such as the case of *Laminaria* (*n* = 10) and *Ulva* (*n* = 8) than in other species (Figure 3).

**Figure 4:**
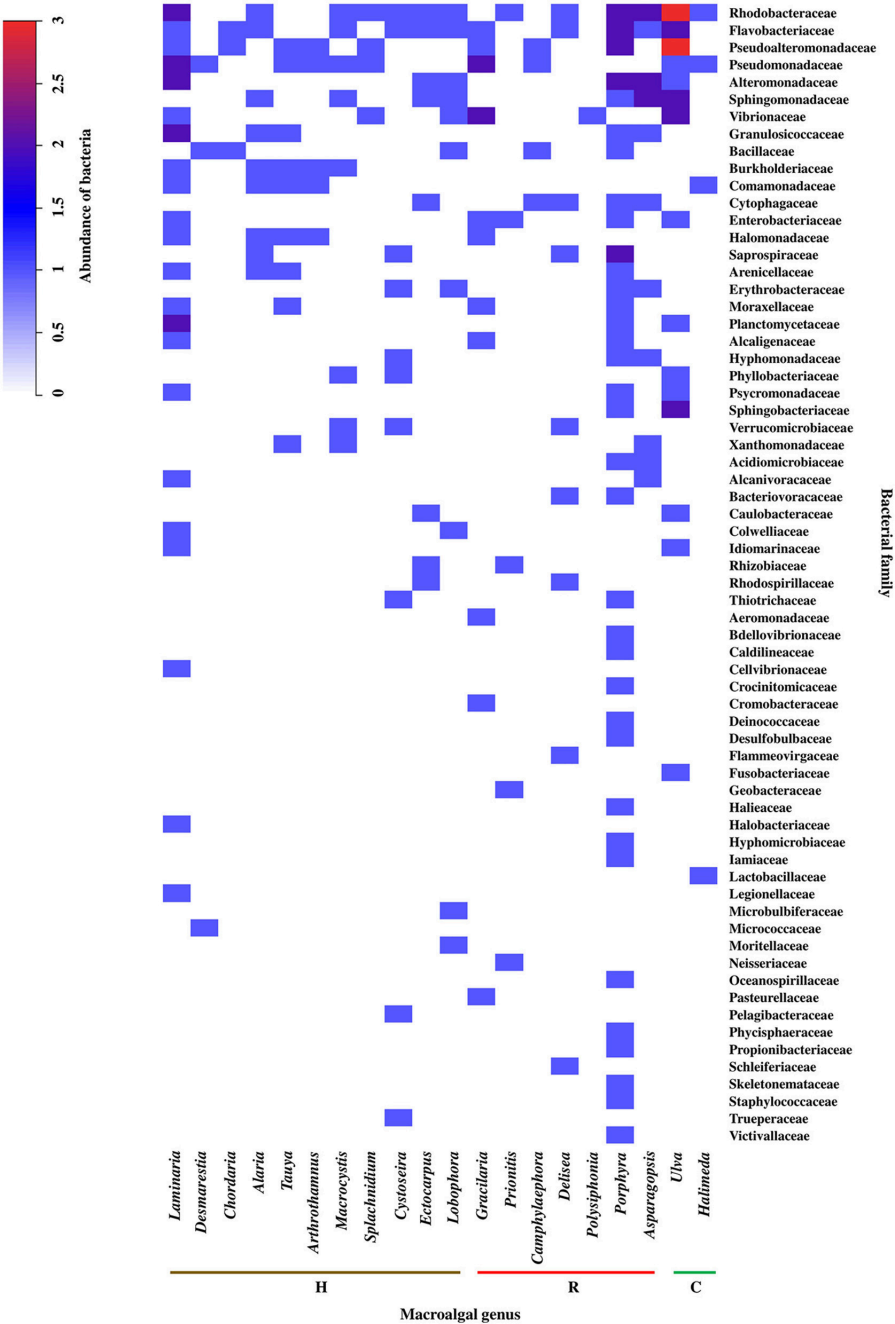
Abundance and distribution of epiphytic bacteria, at the family level, associated to macroalgal genera of the different macroalgal phyla: Chlorophyta (C), Heterokontophyta (H), and Rhodophyta (R). The scale in the upper left shows the correspondence between colors and abundance values of bacteria. Abundance was defined as the number of Presence/Absences of epiphytic bacteria, at the family level, reported for each macroalgal genus in the literature consulted (*n* = 32). The bacterial family were ordered from highest to lowest abundance value. Taxonomic classification corresponded to those used in the literature consulted (Table S5).

Although at the phylum and class level of bacteria, it was possible to identify certain grouping among the macroalgal genera, when analyzing the information in terms of taxonomic assignment of bacteria at the family level, a different grouping pattern was obtained (cut-off point = 30% similitude; Figure S2), but still without discriminating between macroalgal phyla (Figure 4). The highest abundance values were found associated with genera of different macroalgal phyla such as *Pyropia, Asparagopsis* and *Gracilaria* (Rhodophyta), *Laminaria* (Heterokontophyta), and *Ulva* (Chlorophyta). Within these genera, the most abundant bacterial groups were Rhodobacteraceae, Flavobacteriaceae, Pseudoalteromonadaceae, Pseudomonadaceae, Alteromonadaceae, Sphingomonadaceae, Vibrionaceae, Granulosicoccaceae, Saprospiraceae, Planctomycetaceae, and Sphingobacteriaceae. Furthermore, it was observed that, of a total of 66 bacterial families, 18 were associated particularly with *Pyropia*, 7 with *Laminaria*, 3 with *Ulva*, and 1 with *Asparagopsis* (Figure 4).

**Figure 5:**
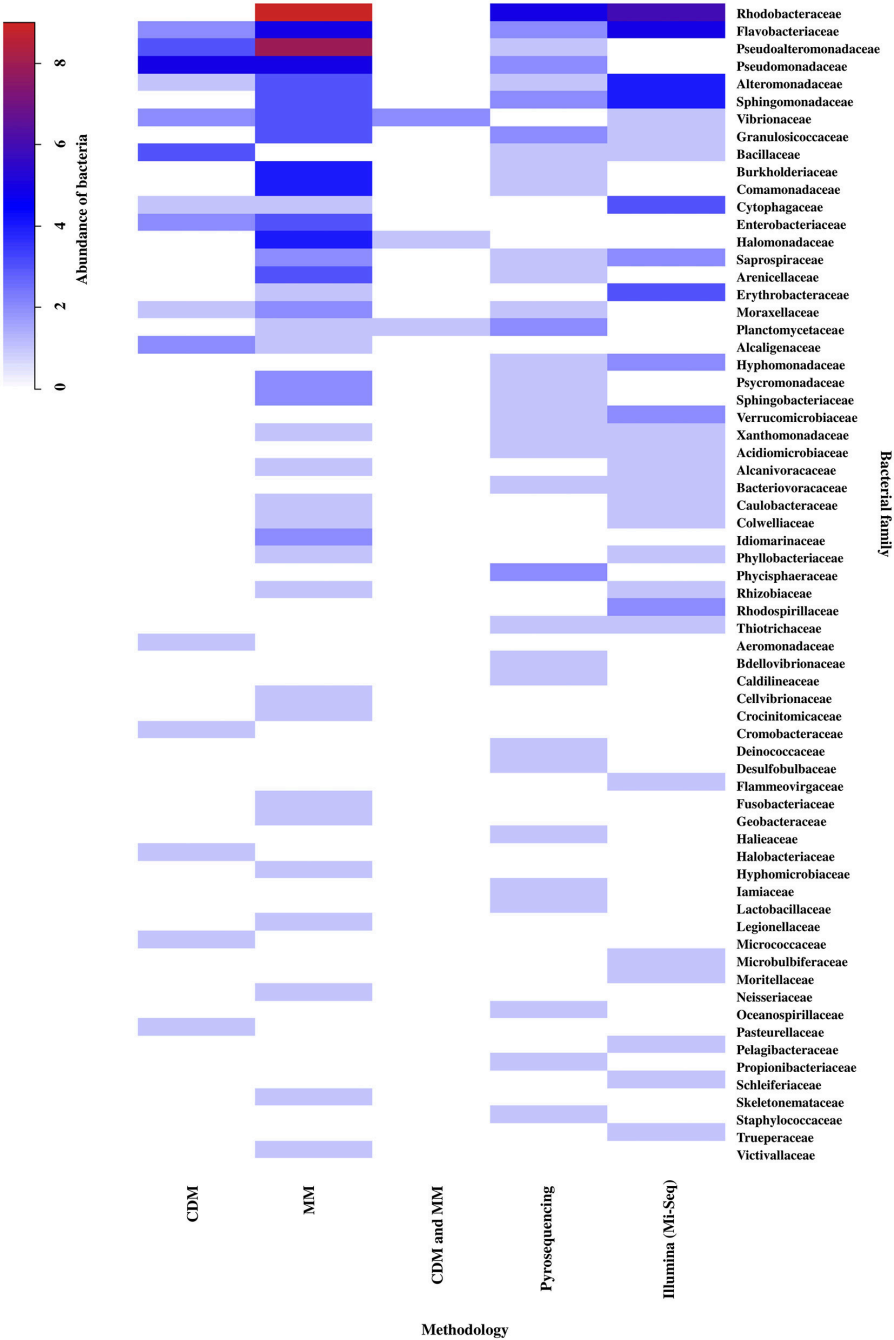
Abundance and distribution of epiphytic bacteria, at the family level, associated to the different methodological approaches: Culture-Dependent Methods (CDM), Molecular Methods (MM), Culture-Dependent Methods and Molecular Methods (CDM + MM), Pyrosequencing and Illumina (Mi-Seq). The scale in the upper left shows the correspondence between colors and abundance values of bacteria. Abundance was defined as the number of Presence/Absences of epiphytic bacteria, at the family level, reported for each methodological approach in the literature consulted (*n* = 32). The bacterial family were ordered from highest to lowest abundance value. Taxonomic classification corresponded to those used in the literature consulted (Table S6).

The methodology used to study the EBCs-macroalgae association was considered an important factor for the analysis of the results, since each one allows obtaining a different resolution for the bacterial taxonomic classifications (Suenaga, 2012). According to this review, the taxonomic resolution is relevant when seeking to differentiate between genera of macroalgae based on their EBCs. At the level of phylum and Proteobacteria class, there was no discrimination between genera of macroalgae (data not shown). However, increasing the degree of taxonomic resolution of bacteria at the family level, formed 3 groups corresponding to the macroalgal phyla (cutoff = 30% similarity; Figure S3). For this reason, the bacterial family level was used to analyze the importance of the methodology in the differentiation of macroalgae EBCs (Figure 5). The results showed that Pyrosequencing was the method detecting a greater number of particular families (11 families), followed by molecular methods (10 families), Illumina (7 families), and to a lesser extent, by culture-dependent methods (5 families). The greatest bacterial abundances were obtained for the families Rhodobacteraceae, Flavobacteriaceae, Pseudoalteromonadaceae, Pseudomonadaceae, Alteromonadaceae, Sphingomonadaceae, Burkholderiaceae, Commamonadaceae, and Halomonadaceae (Figure 5). When grouping by bacterial family, higher similarities were found between molecular methods and Illumina technology (e.g., Mi-Seq), than with other methodologies and, in turn, culture-dependent techniques present least similarity with other methodologies.

**Figure 6:**
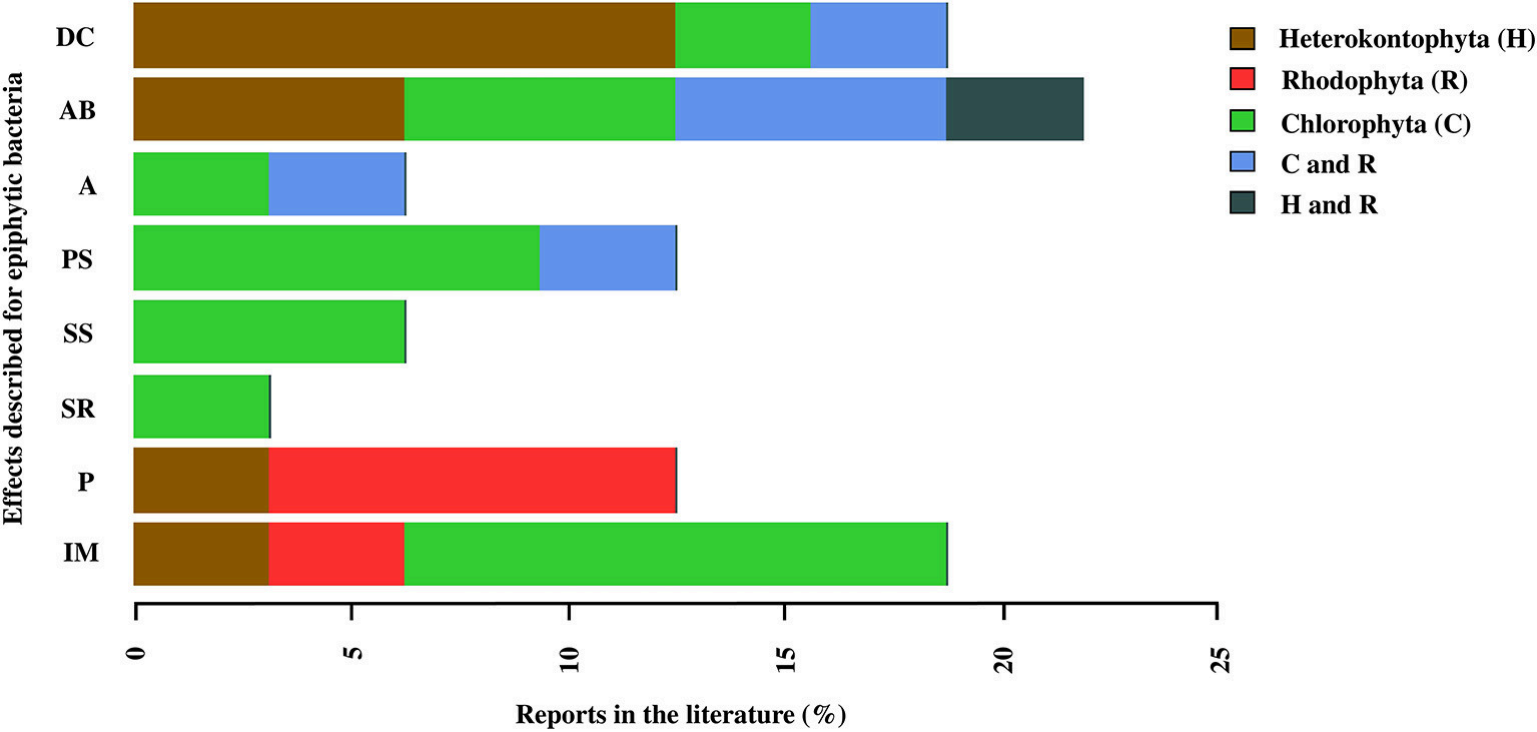
Reports in the literature of the effects associated with epiphytic bacteria in macroalgal phyla: Chlorophyta (C), Heterokontophyta (H), and Rhodophyta (R). The effects correspond to: Inducing morphogenesis (IM), Pathogen (P), Spore release (SR), Stimulation settlement of algal spores (SS), Prevent settlement of algal spores (PS), Antifouling (A), Antibacterial activity and Degradation of algal compounds (DC). The percentage values for each macroalgal phylum were calculated in relation to the total number of papers reviewed (*n* = 25), and the percentages of each effect for each macroalgal phylum were added. The macroalgal phyla were arranged in alphabetical order (Table S7).

Finally, 23 of the studies analyzed in this review, evaluated different functions of the epiphytic bacteria associated with the macroalgae. The results showed that the functions of these bacteria are associated with antibacterial activity (21.8%), degradation of the macroalgal compounds (18.75%), induction of morphogenesis (18.7%), and pathogenic activity (12.5%; Figure 6). However, studies have also focused particularly on some of these effects in the different groups of macroalgae, for example, the effect of degradation of macroalgal compounds in Heterokontophyta, the effects of pathogenic activity or interkingdom cell to cell communication in Rhodophyta and, the bacterial effects on macroalgal morphogenesis and sporulation processes, both in the release induction and settlement, in Chlorophyta (Figure 6).

**Figure 7:**
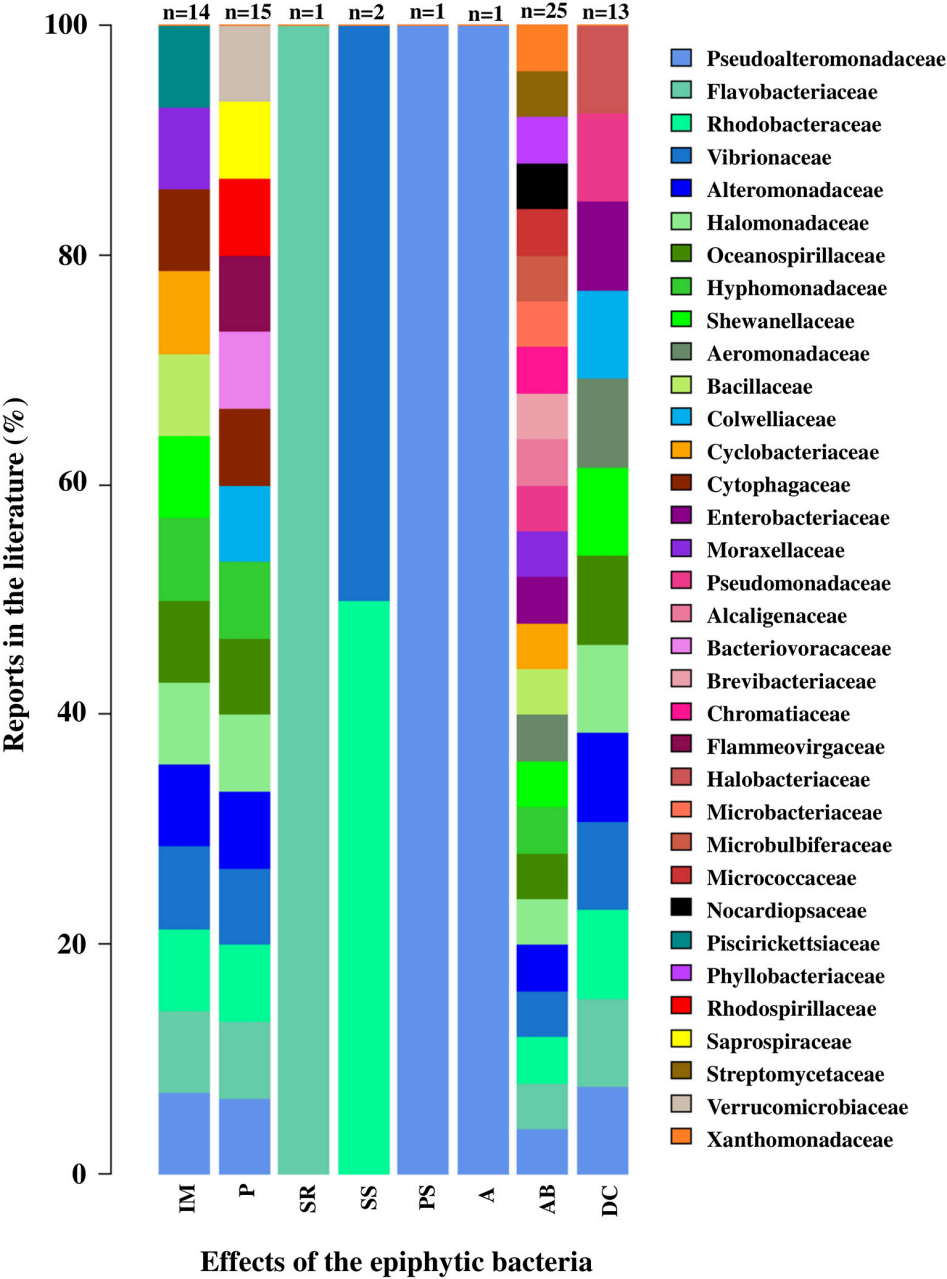
Effects associated with epiphytic bacterial families in macroalgae. The effects correspond to: Inducing morphogenesis (IM), Pathogen (P), Spore release (SR), Stimulation settlement of algal spores (SS), Prevent settlement of algal spores (PS), Antifouling (A), Antibacterial activity (AB) and Degradation of algal compounds (DC). Percentage values for each bacterial family were calculated relative to the number of papers reviewed for each function (*n*-values presented in the graph). The bacterial family were ordered from lowest to highest percentage value. Taxonomic classification corresponded to those used in the literature consulted (Tables S8, S9).

Some of these bacterial functions are general and can be carried out by different families, but others are specific to only one type (Figure 7). Of a total of 34 families of epiphytic bacteria reported, a total of 8 functions were analyzed in this review. Seventeen bacterial families were related to only one function, including Saprospiraceae (pathogenesis), Piscirickettsiaceae (morphogenesis induction), Halobacteriaceae (degradation of macroalgal compounds), and Streptomycetaceae (antibacterial activity). While the families Alteromonadaceae, Halomonoadaceae, Hypomonadaceae, Flavobacteriaceae, Pseudoalteromonadaceae, Rhodobacteraceae, and Vibrionaceae were related to between 4 and up to 6 functions from the ones categorized in this review (Figure 7).

## Discussion

### Differentiation among macroalgae based on their EBC

The first result found in this review showed that the EBC found on the different macroalgal phyla have some differences (Figure 1), but only a low percentage (20% for Rhodophyta, 4% for Heterokontophyta, and none for Chlorophyta) of the bacterial phyla and classes appears to be specific to some macroalgal phyla. The differences could be explained due to the specific cell wall polysaccharides found on each macroalgal phyla (e.g., agar, carrageenan, alginate, ulvans; Popper et al., 2011), and the capability of epiphytic microorganisms to produce specific enzymes for their degradation (Martin et al., 2014). In addition, the production and exudate of secondary metabolites by the different macroalgae can selectively attract or repel some bacteria (Collén and Davison, 2001).

Similar results were reported by Burke et al. (2011b), who demonstrated that *U. australis* does not possess a core bacterial community. Previous research recognizes that the species *U. australis* has relatively few chemical protection mechanisms against colonizers, which could lead to less selectivity with regards to the bacterial diversity in the environment, and increase the importance of bacteria that carry out this protective function (Holmström et al., 2002). Most of the literature on Chlorophyta macroalgae included in our analysis used *U. australis* as a biological model, hence it is possible that this may have contributed to the scarce reports of bacteria specific to this phylum. Therefore, it is expected that the analysis of epiphytic communities of other green macroalgae species, may contribute to establish the presence of bacteria taxa unique to this group, which is currently unknown. In contrast, various studies have shown the antifouling capacity of macroalgae from Heterokontophyta (Borchardt et al., 2001; Saha et al., 2011) and Rhodophyta (Maximilien et al., 1998; Manefield et al., 1999), where chemical interference in bacterial communication mechanisms for biofilm formation has been identified. Some studies such as those carried out by Manefield et al. (1999) and Rasmussen et al. (2000) demonstrated the antifouling activity of *D. pulchra* (Rhodophyta) through the production of furanones. Rasmussen et al. (2000) demonstrated that furanones not only interfere intraspecific cell to cell communication in bacteria, but also interspecific communication. In this sense, furanones reduce bacterial motility inhibiting the serrawettin production, a bacterial surfactant that decrease surface tension for swarming (probably an important process for a successful bacterial colonization on macroalgal surface). In addition, furanones can control bacterial colonization of surfaces by interfering with the mechanism of bacterial communication (e.g., Quorum Sensing), more specifically, with acyl homoserine lactone (AHL) dependent gene transcription at the level of the LuxR like regulatory protein (Manefield et al., 1999; Rasmussen et al., 2000).

Some of the bacterial phyla shared by the three macroalgal groups are Proteobacteria, Bacteroidetes, Verrucomicrobia, Planctomycetes, Firmicutes, and Cyanobacteria, which have frequently been reported as macroalgae-surface associated microorganisms (Bengtsson et al., 2010; Lachnit et al., 2011; Mancuso et al., 2016). While the phyla Chlorobi, Chloroflexi, Deinococcus-Thermus, Fusobacteria, Lentisphaera, and Spirochaetes and the Epsilonproteobacteria class, are included in the least frequent bacteria on the macroalgae surface (Barott et al., 2011). However, some genera from the phylum Chlorobi and Spirochaetes have been described as having cellulose degrading potentials (Berlemont and Martiny, 2013), and according to the results of this review, have been reported using molecular methods and NGS technologies. The wide range of habitats where Epsilonproteobacteria can be found it has also been described, due to their ability to grow under aerobic, microaerobic, or anaerobic conditions; although these have been especially related to extreme environments with suboxic to anoxic conditions (Campbell et al., 2006). This evidence suggests a possible bias depending on the study interest and the methodology used for its detection, which will be discussed later.

At macroalgal genus level, the results showed that there is no clear similarity pattern between macroalgae genera (within the same phylum) with respect to their associated bacterial community. As an example, greater similarity was obtained between *Laminaria* and *Ulva* instead of other genera of their own taxonomic grouping. This contrasts with the findings of Lachnit et al. (2009a), they compared the EBCs of different species of the three macroalgal phyla, and found greater differences in the associated bacteria at the phylum level rather than species level. These results were attributed to different chemical compositions and effectiveness in attraction or deterrent mechanisms specific to each macroalgal phylum (Potin et al., 2002). Nevertheless, the results obtained by Staufenberger et al. (2008) and Bengtsson et al. (2010), among the kelp *S. latissima* and *Laminaria hyperborea*, the most abundant bacteria associated with the former species belonged to the Planctomycetes and Verrucomicrobia phyla, while these groups were notably absent in the latter species. The above suggest that the composition and structure of EBC associated with macroalgae does not depend exclusively on the taxonomic grouping of their hosts, but also to other factors such as the characteristics of the environment, seasonality and the development status of the host, affecting significantly the selection of the EBCs (Bengtsson et al., 2010; Campbell et al., 2015). For the purpose in this review, the analysis was performed at the macroalgal genus level, since some of these have been frequently used as biological models in each of their respective groups (e.g., *Laminaria, Gracilaria*, and *Ulva*).

When discrimination was based on the presence of rare bacteria (at the phylum and class level), total number of macroalgal genera dropped from 24 to 9. This coincides with the proposals by Lynch and Neufeld (2015), indicating that certain bacteria with low representation within the community, may contribute to differentiate the habitats in which they develop.

Among the 9 genera, *Laurencia* and *Lobophora* stand out as having the greatest number of rare bacteria. However, according to the functional redundancy hypothesis (Naeem, 1998), more than one bacterial taxon can carry out a specific function. In other words, it can be a bias trying to establish differences or similarities between macroalgae only based on the presence of rare taxa without considering its functionality.

Although variations in the macroalgae EBCs can be found at different taxonomic levels, there is little distinction in these microorganisms at the higher taxonomic levels (Hollants et al., 2012). Thus, the detection of groups of bacteria allowed us to establish a larger differentiation among the macroalgal genera. The analysis of our information, moving from phylum and class level of bacteria to family level, resulted in a greater differentiation between macroalgal genera. In other words, a greater number of families were assigned to particular macroalgal genus. This information is in accordance to Penesyan et al. (2009), that by comparing the EBCs of the macroalgae *D. pulchra* and *U. australis*, found less overlapping at the bacterial species level than at the phylum level.

### Importance of methodology in differentiating macroalgal EBCs

The methodology used to study macroalgal EBCs can be an important factor in identifying and understanding the diversity and function of bacteria inhabiting the macroalgal surface (Egan et al., 2008). Our results showed that the methodological approximations used in different studies on macroalgal EBCs, provided information with different degrees of resolution. Some bacterial diversity patterns have been identified based on their classification at higher taxonomic levels. However, more information is required at the lower taxonomic levels (e.g., family, genus, species) to establish greater differentiation. This information is validated with the results of this review.

During the 1950's, culture-dependent methods were adopted attempting to determine the bacteria associated with macroalgae. Nevertheless, as the culturable bacteria only correspond to <1% of those present in natural environments (Eilers et al., 2000), the information obtained by these methodologies is insufficient to study the EBC.

Selective culture methods and a very general taxonomic assignment are used, based on morphology and biochemical tests (Hollants et al., 2012). A study carried out by Bengtsson et al. (2011) showed that bacteria isolated through cultures differ from those that dominate the EBC of the Heterokontophyta species *L. hyperborean* under natural conditions. This complicates the fully understanding of the interactions among these organisms as some of the bacteria involved in these interactions could not be detected. The results obtained in this review, show that the number of bacterial families found using culture-dependent methods was lower than those identified with culture-independent methods, which coincides with the previous discussions in this paper. Thus, although it is important to emphasize that culture-dependent methods are still an appropriate methodological approach to characterize the metabolic properties of microorganisms, these are not adequate to understand their function at community level or to establish the occurrence of biological interactions between the host and EBC.

With the subsequent development of molecular methods (e.g., DGGE, T-RFLP's, Clonal analysis) some of the limitations associated with the detection of non-culturable bacteria have been overcome. Nevertheless, this type of approximation creates difficulties regarding taxonomic assignment of bacteria, due to the co-migration of fragments with different sequences (Vallaeys et al., 1997), and the detection of less abundant groups (Muyzer and Smalla, 1998; Douterelo et al., 2014). It is possible to improve the limited sensitivity to detect rare members of the community by hybridization analysis with specific probes for each taxon, but the technique remains limited only to the organisms for which such probes have been developed (Muyzer and Smalla, 1998). In this review, the number of bacterial taxa found with molecular methods, was greater than those identified using cultures, which allowed more precise characterization of the EBCs associated with the macroalgae. Different studies have used these methodologies to generate information, at the phylum level, about some of the factors that can affect the abundance, composition and structure of these microbial communities, such as the macroalgae section (Staufenberger et al., 2008), its phenotype (Balakirev et al., 2012), and the site and conditions inhabited (Hengst et al., 2010; Bengtsson et al., 2012).

Over the past few years, many of the limitations mentioned above have been overcome. The NGS technologies have enabled different phylogenetic studies of the EBCs associated with the macroalgae to be conducted. These studies use DNA obtained directly from environmental samples, with a high degree of resolution and taxonomic assignment, as well as being relatively low in terms of cost and effort (Handelsman, 2004; Douterelo et al., 2014). Among these NGS technologies, the platforms used with greatest frequency are Roche 454 (Pyrosequencing) and Illumina/Solexa (e.g., Mi-Seq or Hi-Seq), although, Illumina is currently replacing Roche 454 as the method of sequencing chosen by most of these studies (Douterelo et al., 2014). Contrary to expectations, when comparing the results obtained with both technologies in our analysis, a greater number of unique families were detected through Pyrosequencing. This finding may be due, in order to the methodology comparisons considered in this study, most of the information found on bacteria corresponded to phylum and family level, which could reduce the capacity to discriminate among the NGS technologies. According to the literature, the sequencing error of the Pyrosequencing and Illumina platforms is comparable (Douterelo et al., 2014), indicating that we can analyze and compare data obtained by both methodologies as performed in this study. Within the families detected, we can highlight Oceanospirillaceae (Pyrosequencing), Rhodospirillaceae, Flammeovirgaceae, and Microbulbiferaceae (Illumina), to which belong some bacteria with functions described in their association with macroalgae, as it will be discussed in the following section. Families with high abundance were also found, mainly belonging to the Proteobacteria phylum, characterized as one of the most commonly reported groups of bacteria within the EBCs of macroalgae in marine environments (Mancuso et al., 2016).

In general, each methodology has its advantages and disadvantages and can provide valuable information depending on the study approach, the level of resolution required, the availability of specialized equipment and the availability of financing (Douterelo et al., 2014). However, due to the possible biases associated with each methodology, the integration of multiple methods to obtain more complex analyzes on microbial diversity in natural environments should be considered (Kozdrój and van Elsas, 2001; Dahllöf, 2002). According to this, Giovannoni and Stingl (2007) suggest that, culture approaches, together with the information provided by the metagenomic analysis, are a powerful combination. On the other hand, cells in culture allow to study the whole organism, instead of trying to infer their physiological characteristics. While the genomic and metagenomic data may reveal the metabolic potential that is not known, metabolic pathways, regulatory circuits, and conservation between cultivable and non-culturable bacteria.

### Importance of the functional focus in analysis of macroalgae-associated EBCs

Compared with the traditional focus of analysis, where the study of macroalgal EBCs was limited to their composition and structure, a perspective based on the holobiont concept has recently emerged, which recognizes that the ecology and development of the macroalgae cannot be understood without considering the interactions with their associated microorganisms (Barott et al., 2011; Egan et al., 2013; Aires et al., 2015; Wichard et al., 2015). The development of NGS technologies has enabled studies with this holistic focus to be conducted, since in addition to facilitating the detection of an enormous microbial diversity, yet still unknown, also allows to generate knowledge that facilitates the understanding of a variety of functions associated with the bacteria in their interaction with the macroalgae. The study of the functional genes, which that can be associated with a specific function, is essential when linking microbial diversity to specific ecological functions. In conformity with the above, different functions carried out by the bacteria associated with macroalgae have been identified. This review showed that most of the studies are focused on evaluating antibacterial activity, which makes sense if we consider that the surface of the macroalgae is an important source of substrate and nutrients, and, as such, can be a highly competitive environment (Armstrong et al., 2001). Other aspects evaluated also include the effects associated with the degradation of macroalgal compounds (e.g., alginate and mannitol, among others; Bengtsson et al., 2011), the induction of macroalgal morphogenesis (Matsuo et al., 2003, 2005; Singh et al., 2011; Spoerner et al., 2012; Grueneberg et al., 2016), pathogenic activity (Wang et al., 2008), antifouling activity (Holmström et al., 2002), and the prevention or stimulation of sporulation and/or settlement of macroalgae spores (Matsuo et al., 2003; Patel et al., 2003; Rao et al., 2007; Singh et al., 2015; Wichard, 2015). In general, some of these functions have been deeply studied in certain macroalgal phyla; however, more than being related to characteristics of the macroalgal groups, these results could be conditioned by the objectives of the studies considered in this review, and the recurrent use of certain biological models. As an example, the case of the species *U. australis* where different authors have suggested a low chemical defense capacity and indicated the importance of identifying the antibacterial potential of its EBC, which could be adopting this role (Egan et al., 2000; Holmström et al., 2002; Goecke et al., 2010). However, studies have not been limited to this group of macroalgae. Wiese et al. (2009) found that 49% of bacteria isolated from *S. latissima* presented antimicrobial activity, which differs from the isolated planktonic bacteria, in which the percentage of bacteria with this potential was significantly lower. Some bacteria reported with antibacterial activity, in the different macroalgal phyla, correspond to members of the Pseudoalteromonadaceae, Pseudomonadaceae, Aeromonadaceae, Alcaligenaceae, Halomonadaceae, Hyphomonadaceae, Micrococcaceae, Streptomycetaceae, families, among others.

Effects of microorganisms on morphological development has been extensively covered in Chlorophyta albeit based on isolated bacteria but not from EBCs. In a pioneering research, Provasoli (1958) demonstrated that “the typical thallus was never obtained in bacteria free cultures in *Ulva* sp.” but also found that *Ulva* responds to plant hormones (e.g., indolacetic acid and kinetins), suggesting that bacterial surrounding probably produce similar molecules, leading algal development. Afterwards, Provasoli and Pintner (1980) observed a loss in normal morphology of *U. lactuca* when it was kept in axenic cultures, and its subsequent recuperation through re-inoculation with previously isolated bacteria from the macroalgae surface. Similar results were reported for the Chlorophyta *Monostroma oxyspermum, U. pertusa, U. conglobate, and U. intestinalis* (Matsuo et al., 2003) and *Ulva fasciata* (Singh et al., 2011), in *Pyropia yezoensis* (Rhodophyta) (Fukui et al., 2014) and in *Ectocarpus* sp. (Heterokontophyta) (Tapia et al., 2016). A well-characterized association between bacteria and macroalgae was described by Matsuo et al. (2003) in *M. oxyspermum*, demonstrating that axenic gametes inoculated with *Roseobacter* sp. MS2 and *Cytophaga* MS6 could induce cell division, cell differentiation and cell wall formation. In the same way, Spoerner et al. (2012), demonstrated that *U. mutabilis* development also depends on a microbial core composed by three Proteobacteria (i.e., *Roseobacter, Sulfitobacter*, and *Halomonas*) and a Bacteroidetes from the *Cytophaga* genus.

Recently, Wichard et al. (2015) propose to Ulvales as a good model species to study inter-kingdoms interactions, based on that they require a symbiosis with bacteria to reach a normal morphogenesis; although the mechanisms have not been completely elucidated. Chemical compounds mediating communication between bacteria or bacteria-alga, has been studied for a longtime; however only two of them have been characterized, acyl homoserine lactone (AHL; Wheeler et al., 2006) and thallusin (from *Cytophaga* sp. strain YM2-23; Matsuo et al., 2005). These compounds exert differential effects on the algal life cycle; for example, AHL from several gram-negative bacteria, attract and induce settlement of *Ulva* zoospores (Joint et al., 2002); and particularly in *U. intestinalis* is mediated by a chemokinetic mechanism (Wheeler et al., 2006). Otherwise, thallusin induced morphogenesis in *M. oxyspermum*, but also stimulated the normal germination in germfree spores from *U. pertusa and U. intestinalis* (Matsuo et al., 2003, 2005).

Interestingly, studies undertaken by Burke et al. (2011a,b) revealed differences in the composition of the EBCs among individuals of the species *U. australis* inhabiting the same site, while the functional composition was very similar. This suggests that for analyzing the composition, structure and interactions of the EBCs associated with the macroalgae, the key level would be the functional bacterial genes rather than bacterial species alone (Burke et al., 2011a).

While Campbell et al. (2015) suggests an integrative vision in which the combination of processes influencing the composition and structure of the EBCs associated with macroalgae is considered. Their results demonstrate that the composition of EBC on *Phyllospora comosa* (Heterokontophyta) is not habitat-specific (effect of local conditions), but host-specific. Therefore, although EBCs may vary between sites depending on available taxa, some bacteria may remain driven by specific host traits.

Since the family was the lowest taxonomic level used to compare the information in the present study, we found some bacteria associated with different functions within the same family, such as the case of Alteromonadaceae, Halomonadaceae, Hypomonadaceae, Flavobacteriaceae, Pseudoalteromonadaceae, Rhodobacteraceae, and Vibrionaceae. Furthermore, these families were detected in the EBCs of macroalgal genus in different phyla by several of the methodologies analyzed, and associated with antimicrobial activity function, one of the most documented functions (Patel et al., 2003; Rao et al., 2005; Penesyan et al., 2009; See Table S7).

In contrast, the families Alcaligenaceae, Microbulbiferaceae, Micrococcaceae, Streptomycetaceae, Verrucomicrobiaceae, Xanthomonadaceae (associated with an antimicrobial activity), Saprospiraceae, Bacteriovoracaceae, Rhodospirillaceae, Flammeovirgaceae (associated with a pathogenic activity), Halobacteriaceae (associated with macroalgal compound degradation), and Piscirickettsiaceae (associated with morphogenesis induction), were only associated with one particular function. They were all found in the EBCs of the Heterokontophyta and Rhodophyta phylum, and some were detected particularly using NGS technologies, which reflects the importance of using high resolution methodologies. Wang et al. (2008) analyzed the culturable epiphytic bacteria associated with a disease of *Saccharina japonica* (formerly *Laminaria japonica*), and found that a high proportion of the isolated bacteria corresponded to the genus *Pseudoalteromonas* (Pseudoalteromonadaceae), which is recognized as producing proteolytic enzymes that can decompose the macroalgal cell wall and cause disease. Nevertheless, on re-infecting algae healthy tissue with isolated bacteria, the disease did not materialize, suggesting that the pathogenic bacteria causing the diseases may belong to the non-culturable fraction of the EBC (Wang et al., 2008).

## Conclusion

This review analyzed the EBCs associated with macroalgae using different taxonomic levels of bacteria and conclude that: (1) The taxonomic grouping of macroalgae does not explain the composition and structure of the EBCs. (2) It is important to distinguish between the methodology used to describe EBCs considering the best characterization of bacterial groups with a higher degree of resolution. (3) Since different bacteria can have the same function, it is important to recognize host-specific and generalist bacteria, to describe the functionality of EBCs. In this review, we recommend the incorporation of a complementary approach between the taxonomic composition and the functional composition analyzes of the EBCs, as well as the use of methodological tools that enable analysis of interactions between the EBCs and their hosts, based on the holobiont concept. Furthermore, given the complexity of macroalgae as a live substrate responding and interacting with its epiphytic bacterial community, the information on the composition and structure of the associated bacterial communities must be complemented with studies focusing on possible responses of macroalgae to functional interactions with their associated microorganisms.

Finally, it is recognized that bacteria can have positive and negative effects on their interaction with macroalgae (Goecke et al., 2010, 2013b). In this review, we included several studies with results showing positive effects of epiphytic bacteria on macroalgae, such as the induction of morphological development (Matsuo et al., 2003; Grueneberg et al., 2016) and the anti-microbial activity (Holmström et al., 2002; Rao et al., 2007; Wang et al., 2009), as well as the negative effects, for example, pathogenic bacteria (Wang et al., 2008; Case et al., 2011; Zozaya-Valdes et al., 2015) and bacteria related to macroalgae degradation (Wang et al., 2008; Martin et al., 2015).

However, from this study it is clear that many of the examples have been studied using only one or very few groups of macroalgae. Thus, for example, the effect of bacteria on the induction of morphogenesis has been extensively studied in green algae (Wichard, 2015), but lesser in brown and red. For this reason, the positive or negative interactions that epiphytic bacteria may establish with macroalgae, are still limited to state generalizations. This makes it necessary to continue carrying out studies to deepen the global understanding of the macroalga-bacteria relationship within the holobiont concept.

## Author contributions

JF was responsible for the acquisition of information; JF, CC, MH, and AB for the analysis and interpretation of data; all the authors contributed to producing the paper.

### Conflict of interest statement

The authors declare that the research was conducted in the absence of any commercial or financial relationships that could be construed as a potential conflict of interest.

## References

[B1] Aires T. Moalic Y. Serrão E. A. Arnaud-Haond S. (2015). Hologenome theory supported by co-occurrence networks of species-specific bacterial communities in siphonous algae (Caulerpa). FEMS Microbiol. Ecol. 91, 1–14. 10.1093/femsec/fiv067 26099965

[B2] Aires T. Serrão E. A. Engelen A. H. (2016). Host and environmental specificity in bacterial communities associated to two highly invasive marine species (genus Asparagopsis). Front. Microbiol. 7:559. 10.3389/fmicb.2016.00559 27148239 PMC4839258

[B3] Albakosh M. A. Naidoo R. K. Kirby B. Bauer R. (2016). Identification of epiphytic bacterial communities associated with the brown alga *Splachnidium rugosum*. J. Appl. Phycol. 28, 1891–1901. 10.1007/s10811-015-0725-z

[B4] Almanza V. Buschmann A. H. Hernández-González M. C. Henríquez L. A. (2012). Can giant kelp (*Macrocystis pyrifera*) forests enhance invertebrate recruitment in southern Chile? Mar. Biol. Res. 8, 855–864. 10.1080/17451000.2012.692159

[B5] Armstrong E. Yan L. Boyd K. G. Wright P. C. Burgess J. G. (2001). The symbiotic role of marine microbes on living surfaces. Hydrobiologia 461, 37–40. 10.1023/A:1012756913566

[B6] Ashen J. B. Goff L. J. (2000). Molecular and ecological evidence for species specificity and coevolution in a group of marine algal-bacterial symbioses. Appl. Environ. Microbiol. 66, 3024–3030. 10.1128/AEM.66.7.3024-3030.2000 10877801 PMC92106

[B7] Balakirev E. S. Krupnova T. N. Ayala F. J. (2012). Symbiotic associations in the phenotypically-diverse brown alga *Saccharina japonica*. PLoS ONE 7:e39587. 10.1371/journal.pone.0039587 22745792 PMC3379999

[B8] Barott K. L. Rodriguez-Brito B. Janouškovec J. Marhaver K. L. Smith J. E. Keeling P.. (2011). Microbial diversity associated with four functional groups of benthic reef algae and the reef-building coral *Montastraea annularis*. Environ. Microbiol. 13, 1192–1204. 10.1111/j.1462-2920.2010.02419.x 21272183

[B9] Beleneva I. A. Zhukova N. V. (2006). Bacterial communities of some brown and red algae from Peter the Great Bay, the Sea of Japan. Microbiology 75, 348–357. 10.1134/S0026261706030180 16871810

[B10] Bengtsson M. Sjøtun K. Lanzén A. Øvreås L. (2012). Bacterial diversity in relation to secondary production and succession on surfaces of the kelp *Laminaria hyperborea*. ISME J. 6, 2188–2198. 10.1038/ismej.2012.67 22763650 PMC3505018

[B11] Bengtsson M. Sjøtun K. Øvreås L. (2010). Seasonal dynamics of bacterial biofilms on the kelp *Laminaria hyperborea*. Aquat. Microb. Ecol. 60, 71–83. 10.3354/ame01409

[B12] Bengtsson M. Sjøtun K. Storesund J. Øvreås L. (2011). Utilization of kelp-derived carbon sources by kelp surface-associated bacteria. Aquat. Microb. Ecol. 62, 191–199. 10.3354/ame01477

[B13] Bengtsson M. M. Øvreås L. (2010). Planctomycetes dominate biofilms on surfaces of the kelp *Laminaria hyperborea*. BMC Microbiol. 10:261. 10.1186/1471-2180-10-261 20950420 PMC2964680

[B14] Berlemont R. Martiny A. C. (2013). Phylogenetic distribution of potential cellulases in bacteria. Appl. Environ. Microb. 79, 1545–1554. 10.1128/AEM.03305-12 23263967 PMC3591946

[B15] Bolinches J. Lemos M. L. Barja J. L. (1988). Population dynamics of heterotrophic bacterial communities associated with *Fucus vesiculosus* and *Ulva rigida* in an estuary. Microb. Ecol. 15, 345–357. 10.1007/BF02012647 24201411

[B16] Borchardt S. A. Allain E. J. Michels J. J. Stearns G. W. Kelly R. F. McCoy W. F. (2001). Reaction of acylated homoserine lactone bacterial signaling molecules with oxidized halogen antimicrobials. Appl. Environ. Microbiol. 67, 3174–3179. 10.1128/AEM.67.7.3174-3179.2001 11425738 PMC92997

[B17] Bulleri F. Benedetti-Cecchi L. Acunto S. Cinelli F. Hawkins S. J. (2002). The influence of canopy algae on vertical patterns of distribution of low-shore assemblages on rocky coasts in the northwest Mediterranean. J. Exp. Mar. Biol. Ecol. 267, 89–106. 10.1016/S0022-0981(01)00361-6

[B18] Burke C. Steinberg P. Rusch D. Kjelleberg S. Thomas T. (2011a). Bacterial community assembly based on functional genes rather than species. Proc. Natl. Acad. Sci. U.S.A. 108, 14288–14293. 10.1073/pnas.1101591108 21825123 PMC3161577

[B19] Burke C. Thomas T. Lewis M. Steinberg P. Kjelleberg S. (2011b). Composition, uniqueness and variability of the epiphytic bacterial community of the green alga *Ulva australis*. ISME J. 5, 590–600. 10.1038/ismej.2010.164 21048801 PMC3105733

[B20] Buschmann A. Correa J. A. Beltran J. Retamales C. A. (1997). Determinants of disease expression and survival of infected individual fronds in wild populations of *Mazzaella laminarioides* (Rhodophyta) in central and southern Chile. Mar. Ecol. Prog. Ser. 154, 269–280. 10.3354/meps154269

[B21] Campbell A. H. Marzinelli E. M. Gelber J. Steinberg P. D. (2015). Spatial variability of microbial assemblages associated with a dominant habitat-forming seaweed. Front. Microbiol. 6:230. 10.3389/fmicb.2015.00230 25859245 PMC4374473

[B22] Campbell B. J. Engel A. S. Porter M. L. Takai K. (2006). The versatile epsilon-proteobacteria: key players in sulphidic habitats. Nat. Rev. Microbiol. 4, 458–468. 10.1038/nrmicro1414 16652138

[B23] Case R. J. Longford S. R. Campbell A. H. Low A. Tujula N. Steinberg P. D.. (2011). Temperature induced bacterial virulence and bleaching disease in a chemically defended marine macroalga. Environ. Microbiol. 13, 529–537. 10.1111/j.1462-2920.2010.02356.x 20946533

[B24] Collén J. Davison I. R. (2001). Seasonality and thermal acclimation of reactive oxygen metabolism in *Fucus vesiculosus* (Phaeophyceae). J. Phycol. 37, 474–481. 10.1046/j.1529-8817.2001.037004474.x

[B25] Croft M. T. Lawrence A. D. Raux-Deery E. Warren M. J. Smith A. G. (2005). Algae acquire vitamin B12 through a symbiotic relationship with bacteria. Nature 438, 90–93. 10.1038/nature04056 16267554

[B26] Dahllöf I. (2002). Molecular community analysis of microbial diversity. Curr. Opin. Biotechnol. 13, 213–217. 10.1016/S0958-1669(02)00314-2 12180095

[B27] de Oliveira L. S. Gregoracci G. B. Silva G. G. Salgado L. T. Filho G. A. Alves-Ferreira M.. (2012). Transcriptomic analysis of the red seaweed *Laurencia dendroidea* (Florideophyceae, Rhodophyta) and its microbiome. BMC Genomics 13:487. 10.1186/1471-2164-13-487 22985125 PMC3534612

[B28] Dittami S. M. Duboscq-Bidot L. Perennou M. Gobet A. Corre E. Boyen C.. (2016). Host–microbe interactions as a driver of acclimation to salinity gradients in brown algal cultures. ISME J. 10, 51–63. 10.1038/ismej.2015.104 26114888 PMC4681850

[B29] Douterelo I. Boxall J. B. Deines P. Sekar R. Fish K. E. Biggs C. A. (2014). Methodological approaches for studying the microbial ecology of drinking water distribution systems. Water Res. 65, 134–156. 10.1016/j.watres.2014.07.008 25105587

[B30] Egan S. Harder T. Burke C. Steinberg P. Kjelleberg S. Thomas T. (2013). Review: the seaweed holobiont: understanding seaweed–bacteria interactions. FEMS Microbiol. 37, 462–476. 10.1111/1574-6976.12011 23157386

[B31] Egan S. Thomas T. Holmström C. Kjelleberg S. (2000). Phylogenetic relationship and antifouling activity of bacterial epiphytes from the marine alga *Ulva lactuca*. Environ. Microbiol. 2, 343–347. 10.1046/j.1462-2920.2000.00107.x 11200436

[B32] Egan S. Thomas T. Kjelleberg S. (2008). Unlocking the diversity and biotechnological potential of marine surface associated microbial communities. Curr. Opin. Microbiol. 11, 219–225. 10.1016/j.mib.2008.04.001 18524668

[B33] Eilers H. Pernthaler J. Glöckner F. O. Amann R. (2000). Culturability and *in situ* abundance of pelagic bacteria from the North Sea. Appl. Environ. Microbiol. 66, 3044–3051. 10.1128/AEM.66.7.3044-3051.2000 10877804 PMC92109

[B34] Fernandes N. Steinberg P. Rusch D. Kjelleberg S. Thomas T. (2012). Community structure and functional gene profile of bacteria on healthy and diseased thalli of the red seaweed *Delisea pulchra*. PloS ONE 7:e50854. 10.1371/journal.pone.0050854 23226544 PMC3513314

[B35] Fisher M. M. Wilcox L. W. Graham L. E. (1998). Molecular characterization of epiphytic bacterial communities on Charophycean green algae. Appl. Environ. Microb. 64, 4384–4389. 9797295 10.1128/aem.64.11.4384-4389.1998 PMC106657

[B36] Fraschetti S. Terlizzi A. Bevilacqua S. Boero F. (2006). The distribution of hydroids (Cnidaria, Hydrozoa) from micro- to macro-scale: spatial patterns on habitat-forming algae. J. Exp. Mar. Biol. Ecol. 339, 148–158. 10.1016/j.jembe.2006.07.007

[B37] Friedrich M. W. (2012). Bacterial communications on macroalgae, in Seaweed Biology, eds Wiencke C. Bischof K. (Heidelberg: Springer), 189–201.

[B38] Fukui Y. Abe M. Kobayashi M. Yano Y. Satomi M. (2014). Isolation of Hyphomonas strains that induce normal morphogenesis in protoplasts of the marine red alga *Pyropia yezoensis*. Microb. Ecol. 68, 556–566. 10.1007/s00248-014-0423-4 24840921

[B39] Giovannoni S. Stingl U. (2007). The importance of culturing bacterioplankton in the “omics” age. Nat. Rev. Microbiol. 5, 820–826. 10.1038/nrmicro1752 17853909

[B40] Goecke F. Labes A. Wiese J. Imhoff J. F. (2010). Chemical interactions between marine macroalgae and bacteria. Mar. Ecol. Prog. Ser. 409, 267–300. 10.3354/meps08607

[B41] Goecke F. Labes A. Wiese J. Imhoff J. F. (2013a). Phylogenetic analysis and antibiotic activity of bacteria isolated from the surface of two co-occurring macroalgae from the Baltic Sea. Eur. J. Phycol. 48, 47–60. 10.1080/09670262.2013.767944

[B42] Goecke F. Thiel V. Wiese J. Labes A. Imhoff J. F. (2013b). Algae as an important environment for bacteria–phylogenetic relationships among new bacterial species isolated from algae. Phycologia 52, 14–24. 10.2216/12-24.1

[B43] Grossart H. P. (2010). Ecological consequences of bacterioplankton lifestyles: changes in concepts are needed. Environ. Microbiol. Rep. 2, 706–714. 10.1111/j.1758-2229.2010.00179.x 23766274

[B44] Grueneberg J. Engelen A. H. Costa R. Wichard T. (2016). Macroalgal morphogenesis induced by waterborne compounds and bacteria in coastal seawater. PLoS ONE 11:e0146307. 10.1371/journal.pone.0146307 26745366 PMC4720170

[B45] Handelsman J. (2004). Metagenomics: application of genomics to uncultured microorganisms. Microbiol. Mol. Biol. Rev. 68, 669–685. 10.1128/MMBR.68.4.669-685.2004 15590779 PMC539003

[B46] Hengst M. B. Andrade S. González B. Correa J. A. (2010). Changes in epiphytic bacterial communities of intertidal seaweeds modulated by host, temporality, and copper enrichment. Microb. Ecol. 60, 282–290. 10.1007/s00248-010-9647-0 20333374

[B47] Hollants J. Leliaert F. De Clerck O. Willems A. (2012). Review: what we can learn from sushi: a review on seaweed–bacterial associations. FEMS Microbiol. Ecol. 83, 1–16. 10.1111/j.1574-6941.2012.01446.x 22775757

[B48] Holmström C. Egan S. Franks A. McCloy S. Kjelleberg S. (2002). Antifouling activities expressed by marine surface associated Pseudoalteromonas species. FEMS Microbiol. Ecol. 41, 47–58. 10.1016/S0168-6496(02)00239-8 19709238

[B49] Ivanova E. P. Bakunina I. Y. Sawabe T. Hayashi K. Alexeeva Y. V. Zhukova N. V.. (2002). Two species of culturable bacteria associated with degradation of brown algae *Fucus evanescens*. Microb. Ecol. 43, 242–249. 10.1007/s00248-001-1011-y 12023731

[B50] Jaffray A. E. Anderson R. J. Coyne V. E. (1997). Investigation of bacterial epiphytes of the agar-producing red seaweed *Gracilaria gracilis* (Stackhouse) Steentoft, Irvine et Farnham from Saldanha Bay, South Africa and Lüderitz, Namibia. Bot. Mar. 40, 569–576. 10.1515/botm.1997.40.1-6.569

[B51] Jensen P. R. Kauffman C. A. Fenical W. (1996). High recovery of culturable bacteria from the surfaces of marine algae. Mar. Biol. 126, 1–7. 10.1007/BF00571371

[B52] Joint I. Tait K. Callow M. E. Callow J. A. Milton D. Williams P.. (2002). Cell-to-Cell communication across the prokaryote eukaryote boundary. Science 298, 1207. 10.1126/science.1077075 12424372

[B53] Kanagasabhapathy M. Sasaki H. Haldar S. Yamasaki S. Nagata S. (2006). Antibacterial activities of marine epibiotic bacteria isolated from brown algae of Japan. Ann. Microb. 56, 167–173. 10.1007/BF03175000

[B54] Kozdrój J. van Elsas J. D. (2001). Structural diversity of microorganisms in chemically perturbed soil assessed by molecular and cytochemical approaches. J. Microb. Methods 43, 197–212. 10.1016/S0167-7012(00)00197-4 11118654

[B55] Lachnit T. Blümel M. Imhoff J. Wahl M. (2009a). Specific epibacterial communities on macroalgae: phylogeny matters more than habitat. Aquat. Biol. 5, 181–186. 10.3354/ab00149

[B56] Lachnit T. Meske D. Wahl M. Harder T. Schmitz R. (2011). Epibacterial community patterns on marine macroalgae are host-specific but temporally variable. Environ. Microbiol. 13, 655–665. 10.1111/j.1462-2920.2010.02371.x 21078035

[B57] Lachnit T. Wahl M. Harder T. (2009b). Isolated thallus-associated compounds from the macroalga *Fucus vesiculosus* mediate bacterial surface colonization in the field similar to that on the natural alga. Biofouling 26, 247–255. 10.1080/08927010903474189 20054721

[B58] Lam C. Stang A. Harder T. (2008). Planktonic bacteria and fungi are selectively eliminated by exposure to marine macroalgae in close proximity. FEMS Microbiol. Ecol. 63, 283–291. 10.1111/j.1574-6941.2007.00426.x 18194343

[B59] Laycock R. A. (1974). The detrital food chain based on seaweeds. I. Bacteria associated with the surface of Laminaria fronds. Mar. Biol. 25, 223–231. 10.1007/BF00394968

[B60] Liu M. Dong Y. Zhao Y. Zhang G. Zhang W. Xiao T. (2011). Structures of bacterial communities on the surface of *Ulva prolifera* and in seawaters in an Ulva blooming region in Jiaozhou Bay, China. World J. Microbiol. Biotechnol. 27, 1703–1712. 10.1007/s11274-010-0627-9

[B61] Longford S. R. Tujula N. A. Crocetti G. R. Holmes A. J. Holmström C. Kjelleberg S. (2007). Comparisons of diversity of bacterial communities associated with three sessile marine eukaryotes. Aquat. Microb. Ecol. 48, 217–229. 10.3354/ame048217

[B62] Lu K. Lin W. Liu J. (2008). The characteristics of nutrient removal and inhibitory effect of *Ulva clathrata* on *Vibrio anguillarum* 65. J. Appl. Phycol. 20, 1061–1068. 10.1007/s10811-007-9307-z

[B63] Luo C. Tsementzi D. Kyrpides N. Read T. Konstantinidis K. T. (2012). Direct comparisons of Illumina vs. Roche 454 sequencing technologies on the same microbial community DNA sample. PLoS ONE 7:e30087. 10.1371/journal.pone.0030087 22347999 PMC3277595

[B64] Lynch M. D. Neufeld J. D. (2015). Ecology and exploration of the rare biosphere. Nat. Rev. Microbiol. 13, 217–229. 10.1038/nrmicro3400 25730701

[B65] Ma Y. Liu P. Yu S. Li D. Cao S. (2009). Inhibition of common fouling organisms in mariculture by epiphytic bacteria from the surfaces of seaweeds and invertebrates. Acta Ecol. Sin. 29, 222–226. 10.1016/j.chnaes.2009.08.004

[B66] Mancuso F. P. D'Hondt S. Willems A. Airoldi L. De Clerck O. (2016). Diversity and temporal dynamics of the epiphytic bacterial communities associated with the canopy-forming seaweed *Cystoseira compressa* (Esper) Gerloff and Nizamuddin. Front. Microbiol. 7:476. 10.3389/fmicb.2016.00476 27092130 PMC4824759

[B67] Manefield M. de Nys R. Naresh K. Roger R. Givskov M. Steinberg P.. (1999). Evidence that halogenated furanones from *Delisea pulchra* inhibit acylated homoserine lactone (AHL)-mediated gene expression by displacing the AHL signal from its receptor protein. Microbiology 145, 283–291. 10.1099/13500872-145-2-283 10075410

[B68] Martin M. Barbeyron T. Martin R. Portetelle D. Michel G. Vandenbol M. (2015). The cultivable surface microbiota of the brown alga *Ascophyllum nodosum* is enriched in macroalgal-polysaccharide-degrading bacteria. Front. Microbiol. 6:1487. 10.3389/fmicb.2015.01487 26734000 PMC4690005

[B69] Martin M. Portetelle D. Michel G. Vandenbol M. (2014). Microorganisms living on macroalgae: diversity, interactions, and biotechnological applications. Appl. Microbiol. Biotechnol. 98, 2917–2935. 10.1007/s00253-014-5557-2 24562178

[B70] Matsuo Y. Imagawa H. Nishizawa M. Shizuri Y. (2005). Isolation of an algal morphogenesis inducer from a marine bacterium. Science 307, 1598–1598. 10.1126/science.1105486 15761147

[B71] Matsuo Y. Suzuki M. Kasai H. Shizuri Y. Harayama S. (2003). Isolation and phylogenetic characterization of bacteria capable of inducing differentiation in the green alga *Monostroma oxyspermum*. Environ. Microbiol. 5, 25–35. 10.1046/j.1462-2920.2003.00382.x 12542710

[B72] Maximilien R. de Nys R. Holmström C. Gram L. Givskov M. Crass K. (1998). Chemical mediation of bacterial surface colonization by secondary metabolites from the red alga *Delisea pulchra*. Aquat. Microb. Ecol. 15, 233–246. 10.3354/ame015233

[B73] Meusnier I. Olsen J. L. Stam W. T. Destombe C. Valero M. (2001). Phylogenetic analyses of *Caulerpa taxifolia* (Chlorophyta) and of its associated bacterial microflora provide clues to the origin of the Mediterranean introduction. Mol. Ecol. 10, 931–946. 10.1046/j.1365-294X.2001.01245.x 11348502

[B74] Michelou V. K. Caporaso J. G. Knight R. Palumbi S. R. (2013). The ecology of microbial communities associated with *Macrocystis pyrifera*. PLoS ONE 8:e67480. 10.1371/journal.pone.0067480 23840715 PMC3686729

[B75] Miranda L. N. Hutchison K. Grossman A. R. Brawley S. H. (2013). Diversity and abundance of the bacterial community of the red macroalga Porphyra umbilicalis: did bacterial farmers produce macroalgae? PLoS ONE 8:e58269. 10.1371/journal.pone.0058269 23526971 PMC3603978

[B76] Morán A. C. Hengst M. B. De la Iglesia R. Andrade S. Correa J. A. González B. (2008). Changes in bacterial community structure associated with coastal copper enrichment. Environ. Toxicol. Chem. 27, 2239–2245. 10.1897/08-112.1 18522451

[B77] Muyzer G. Smalla K. (1998). Application of denaturing gradient gel electrophoresis (DGGE) and temperature gradient gel electrophoresis (TGGE) in microbial ecology. Antonie Van Leeuwenhoek 73, 127–141. 10.1023/A:1000669317571 9602286

[B78] Naeem S. (1998). Species redundancy and ecosystem reliability. Conserv. Biol. 12, 39–45. 10.1046/j.1523-1739.1998.96379.x

[B79] Namba A. Shigenobu Y. Kobayashi M. Kobayashi T. Oohara I. (2010). A new primer for 16S rDNA analysis of microbial communities associated with *Porphyra yezoensis*. Fish. Sci. 76, 873–878. 10.1007/s12562-010-0273-z

[B80] Nylund G. M. Cervin G. Persson F. Hermansson M. Steinberg P. D. Pavia H. (2008). Seaweed defense against bacteria: a poly-brominated 2-heptanone from the red alga *Bonnemaisonia hamifera* inhibits bacterial colonization. Mar. Ecol. Prog. Ser. 369, 39–50. 10.3354/meps07577

[B81] Oliveros J. C. (2007). Venny. An Interactive Tool for Comparing Lists with Venn's Diagrams. Available online at: http://bioinfogp.cnb.csic.es/tools/venny/index.html

[B82] Patel P. Callow M. E. Joint I. Callow J. A. (2003). Specificity in the settlement–modifying response of bacterial biofilms towards zoospores of the marine alga Enteromorpha. Environ. Microbiol. 5, 338–349. 10.1046/j.1462-2920.2003.00407.x 12713460

[B83] Penesyan A. Marshall-Jones Z. Holmstrom C. Kjelleberg S. Egan S. (2009). Antimicrobial activity observed among cultured marine epiphytic bacteria re£ects their potential as a source of new drugs. FEMS Microbiol. Ecol. 69, 113–124. 10.1111/j.1574-6941.2009.00688.x 19453738

[B84] Penhale P. A. Capone D. G. (1981). Primary productivity and nitrogen fixation in two macroalgae-cyanobacteria associations. B. Mar. Sci. 31, 164–169.

[B85] Popper Z. A. Michel G. Hervé C. Domozych D. S. Willats W. G. Tuohy M. G.. (2011). Evolution and diversity of plant cell walls: from algae to flowering plants. Annu. Rev. Plant Biol. 62, 567–590. 10.1146/annurev-arplant-042110-103809 21351878

[B86] Potin P. Bouarab K. Salaün J. P. Pohnert G. Kloareg B. (2002). Biotic interactions of marine algae. Curr. Opin. Plant. Biol. 5, 308–317. 10.1016/S1369-5266(02)00273-X 12179964

[B87] Provasoli L. (1958). Effect of plant hormones in Ulva. Biol. Bull. 114, 375–384. 10.2307/1538992

[B88] Provasoli L. Pintner I. J. (1980). Bacteria induced polymorphism in an axenic laboratory strain of *Ulva lactuca* (Chlorophyceae). J. Phycol. 16, 196–201. 10.1111/j.1529-8817.1980.tb03019.x

[B89] Rao D. Webb J. S. Holmström C. Case R. Low A. Steinberg P.. (2007). Low densities of epiphytic bacteria from the marine alga *Ulva australis* inhibit settlement of fouling organisms. Appl. Environ. Microbiol. 73, 7844–7852. 10.1128/AEM.01543-07 17965210 PMC2168146

[B90] Rao D. Webb J. S. Kjelleberg S. (2005). Competitive interactions in mixed-species biofilms containing the marine bacterium *Pseudoalteromonas tunicata*. Appl. Environ. Microbiol. 71, 1729–1736. 10.1128/AEM.71.4.1729-1736.2005 15811995 PMC1082554

[B91] Rasmussen T. B. Manefield M. Andersen J. B. Eberl L. Anthoni U. Christophersen C.. (2000). How *Delisea pulchra* furanones affect quorum sensing and swarming motility in *Serratia liquefaciens* MG1. Microbiology 146, 3237–3244. 10.1099/00221287-140-12-3237 11101681

[B92] Rosenberg E. Sharon G. Atad I. Zilber-Rosenberg I. (2010). The evolution of animals and plants via symbiosis with microorganisms. Environ. Microbiol. Rep. 2, 500–506. 10.1111/j.1758-2229.2010.00177.x 23766221

[B93] Saha M. Rempt M. Grosser K. Pohnert G. Weinberger F. (2011). Surface-associated fucoxanthin mediates settlement of bacterial epiphytes on the rockweed *Fucus vesiculosus*. Biofouling 27, 423–433. 10.1080/08927014.2011.580841 21547758

[B94] Singh R. P. Baghel R. S. Reddy C. R. K. Jha B. (2015). Effect of quorum sensing signals produced by seaweed-associated bacteria on carpospores liberation from *Gracilaria dura*. Front. Plant Sci. 6:117 10.3389/fpls.2015.00117 25788899 PMC4349058

[B95] Singh R. P. Mantri V. A. Reddy C. R. K. Jha B. (2011). Isolation of seaweed-associated bacteria and their morphogenesis-inducing capability in axenic cultures of the green alga *Ulva fasciata*. Aquat. Biol. 12, 13–21. 10.3354/ab00312

[B96] Spoerner M. Wichard T. Bachhuber T. Stratmann J. Oertel W. (2012). Growth and thallus morphogenesis of *Ulva mutabilis* (Chlorophyta) depends on a combination of two bacterial species excreting regulatory factors. J. Phycol. 48, 1433–1447. 10.1111/j.1529-8817.2012.01231.x 27009994

[B97] Staufenberger T. Thiel V. Wiese J. Imhoff J. (2008). Phylogenetic analysis of bacteria associated with *Laminaria saccharina*. FEMS Microbiol. Ecol. 64, 65–77. 10.1111/j.1574-6941.2008.00445.x 18328081

[B98] Suenaga H. (2012). Targeted metagenomics: a high-resolution metagenomics approach for specific gene clusters in complex microbial communities. Environ. Microbiol. 14, 13–22. 10.1111/j.1462-2920.2011.02438.x 21366818

[B99] Tait K. Joint I. Daykin M. Milton D. L. Williams P. Cámara M. (2005). Disruption of quorum sensing in seawater abolishes attraction of zoospores of the green alga Ulva to bacterial biofilms. Environ. Microbiol. 7, 229–240. 10.1111/j.1462-2920.2004.00706.x 15658990

[B100] Tapia J. E. González B. Goulitquer S. Potin P. Correa J. A. (2016). Microbiota influences morphology and reproduction of the brown alga Ectocarpus sp. Front. Microbiol. 7:197. 10.3389/fmicb.2016.00197 26941722 PMC4765120

[B101] Tujula N. A. Crocetti G. R. Burke C. Thomas T. Holmström C. Kjelleberg S. (2010). Variability and abundance of the epiphytic bacterial community associated with a green marine *Ulvacean alga*. ISME J. 4, 301–311. 10.1038/ismej.2009.107 19829319

[B102] Vairappan C. S. Suzuki M. (2000). Dynamics of total surface bacteria and bacterial species counts during desiccation in the Malaysian sea lettuce, *Ulva reticulata* (Ulvales, Chlorophyta). Phycol. Res. 48, 55–61. 10.1111/j.1440-1835.2000.tb00197.x

[B103] Vairappan C. S. Suzuki M. Motomura T. Ichimura T. (2001). Pathogenic bacteria associated with lesions and thallus bleaching symptoms in the Japanese kelp *Laminaria religiosa* Miyabe (Laminariales, Phaeophyceae). Hydrobiologia 445, 183–191. 10.1023/A:1017517832302

[B104] Vallaeys T. Topp E. Muyzer G. Macheret V. Laguerre G. Rigaud A. (1997). Evaluation of denaturing gradient gel electrophoresis in the detection of 16S rDNA sequence variation in rhizobia and methanotrophs. FEMS Microbiol. Ecol. 24, 279–285. 10.1111/j.1574-6941.1997.tb00445.x

[B105] Vieira C. Engelen A. H. Guentas L. Aires T. Houlbreque F. Gaubert J.. (2016). Species specificity of bacteria associated to the brown seaweeds Lobophora (Dictyotales, Phaeophyceae) and their potential for induction of rapid coral bleaching in *Acropora muricata*. Front. Microbiol. 7:316. 10.3389/fmicb.2016.00316 27047453 PMC4800410

[B106] Wahl M. Goecke F. Labes A. Dobretsov S. Weinberger F. (2012). Review: the second skin: ecological role of epibiotic biofilms on marine organisms. Aquat. Microbiol. 292, 1–21. 10.3389/fmicb.2012.00292 PMC3425911 22936927

[B107] Wang G. Shuai L. Li Y. Lin W. Zhao X. Duan D. (2008). Phylogenetic analysis of epiphytic marine bacteria on hole-rotten diseased sporophytes of *Laminaria japonica*. J. Appl. Phycol. 20, 403–409. 10.1007/s10811-007-9274-4

[B108] Wang Z. Xiao T. Pang S. Liu M. Yue H. (2009). Isolation and identification of bacteria associated with the surfaces of several algal species. Chin. J. Oceanol. Limn. 27, 487–492. 10.1007/s00343-009-9165-4

[B109] Wheeler G. L. Tait K. Taylor A. Brownlee C. Joint I. (2006). Acyl-homoserine lactones modulate the settlement rate of zoospores of the marine alga *Ulva intestinalis* via a novel chemokinetic mechanism. Plant Cell Environ. 29, 608–618. 10.1111/j.1365-3040.2005.01440.x 17080611

[B110] Whitman W. B. Coleman D. C. Wiebe W. J. (1998). Prokaryotes: the unseen majority. Proc. Natl. Acad. Sci. U.S.A. 95, 6578–6583. 10.1073/pnas.95.12.6578 9618454 PMC33863

[B111] Wichard T. (2015). Exploring bacteria-induced growth and morphogenesis in the green macroalga order Ulvales (Chlorophyta). Front. Plant. Sci. 6:86. 10.3389/fpls.2015.00086 25784916 PMC4347444

[B112] Wichard T. Charrier B. Mineur F. Bothwell J. H. De Clerck O. Coates J. C. (2015). The green seaweed Ulva: a model system to study morphogenesis. Front. Plant Sci. 6:72. 10.3389/fpls.2015.00072 25745427 PMC4333771

[B113] Wiese J. Thiel V. Nagel K. Staufenberger T. Imhoff J. F. (2009). Diversity of antibiotic-active bacteria associated with the brown alga *Laminaria saccharina* from the Baltic Sea. Mar. Biotechnol. 11, 287–300. 10.1007/s10126-008-9143-4 18855068

[B114] Yarza P. Yilmaz P. Pruesse E. Glöckner F. O. Ludwig W. Schleifer K. H.. (2014). Uniting the classification of cultured and uncultured bacteria and archaea using 16S rRNA gene sequences. Nat. Rev. Microbiol. 12, 635–645. 10.1038/nrmicro3330 25118885

[B115] Zhang Y. Ling J. Yang Q. Wen C. Yan Q. Sun H.. (2015). The functional gene composition and metabolic potential of coral-associated microbial communities. Sci. Rep. 5:16191. 10.1038/srep16191 26536917 PMC4633650

[B116] Zozaya-Valdes E. Egan S. Thomas T. (2015). A comprehensive analysis of the microbial communities of healthy and diseased marine macroalgae and the detection of known and potential bacterial pathogens. Front. Microbiol. 6:146. 10.3389/fmicb.2015.00146 25759688 PMC4338804

