# Supplementary material for: A Functional Perspective Analysis of Macroalgae and Epiphytic Bacterial Community Interaction
Source: Front Microbiol. 2017 Dec 22;8:2561. doi: 10.3389/fmicb.2017.02561 (PMC5743738; doi:10.3389/fmicb.2017.02561)
Supplement: Supplementary file 10 [file Image1.PDF]

SUPPLEMENTARY MATERIAL

Figure S1. Cluster analysis of the epiphytic bacteria at the phylum level, associated to macroalgal genera of the different macroalgal phyla~~present in the different genus of macroalgae~~. The dissimilarity analysis is based on the Bray-Curtis index and ~~to establish differences between the three groups of algae,~~ a cut-off point of ~~4~~55% dissimilarity was established. Taxonomic classification~~The scientific names~~ correspond to those used in the literature consulted (Table S2).

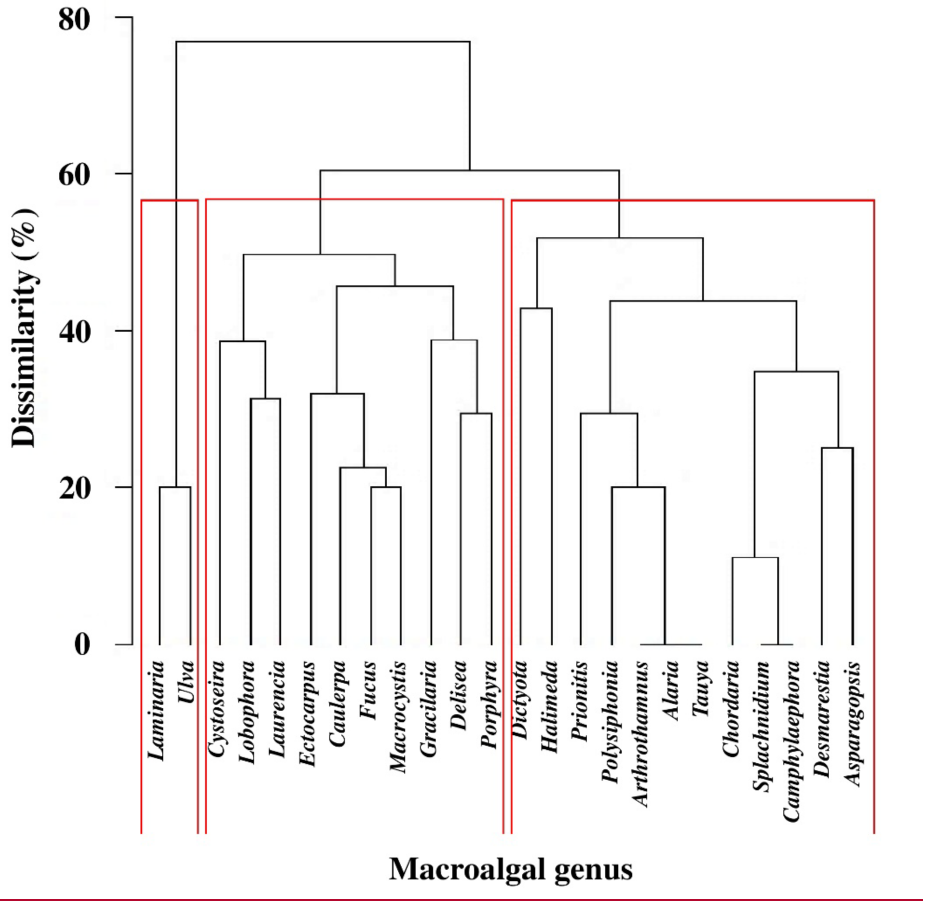

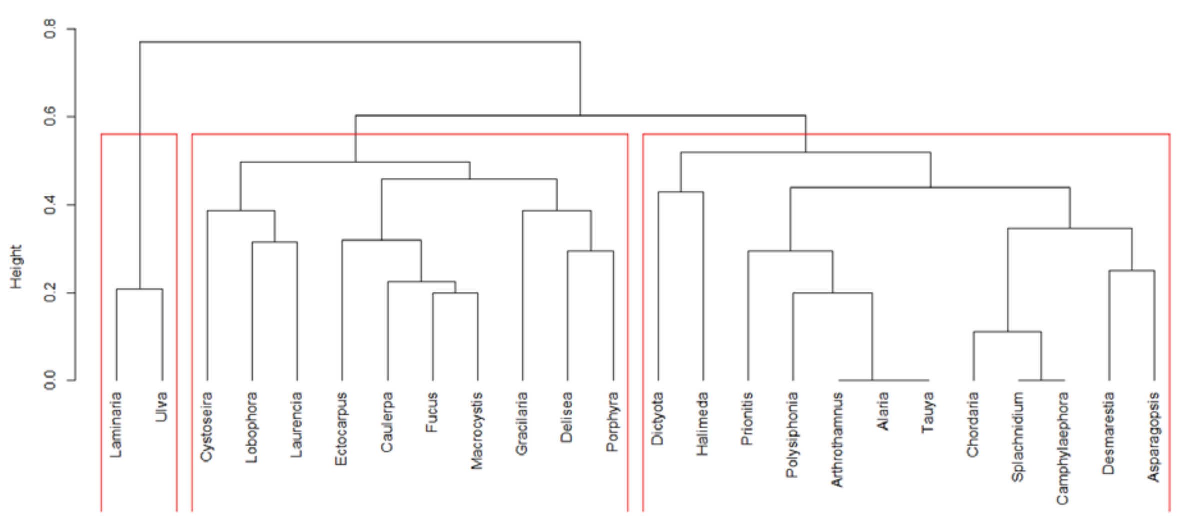
Height
0.0
0.2
0.4
0.6
0.8
Laminaria
Ulva
Cystoseira
Lobophora
Laurencia
Ectocarpus
Caulerpa
Fucus
Macrocystis
Gracilaria
Delisea
Porphyra
Dictyota
Halimeda
Prionitis
Polysiphonia
Arthrothamnus
Alaria
Tauya
Chordaria
Splachnidium
Camphylaephora
Desmarestia
Asparagopsis

1
