# Supplementary material for: A Functional Perspective Analysis of Macroalgae and Epiphytic Bacterial Community Interaction
Source: Front Microbiol. 2017 Dec 22;8:2561. doi: 10.3389/fmicb.2017.02561 (PMC5743738; doi:10.3389/fmicb.2017.02561)
Supplement: Supplementary file 11 [file Image2.PDF]

SUPPLEMENTARY MATERIAL

Figure S2. Cluster analysis of the epiphytic bacteria at the family level, associated to macroalgal genera of the different macroalgal phyla~~present in the different genus of macroalgae~~. The dissimilarity analysis is based on the Bray-Curtis index and a cut-off point of 8~~3~~0% dissimilarity was established~~applied to establish differences between the three groups of algae~~. Taxonomic classification ~~The scientific names~~ correspond to those used in the literature consulted (Table S4).

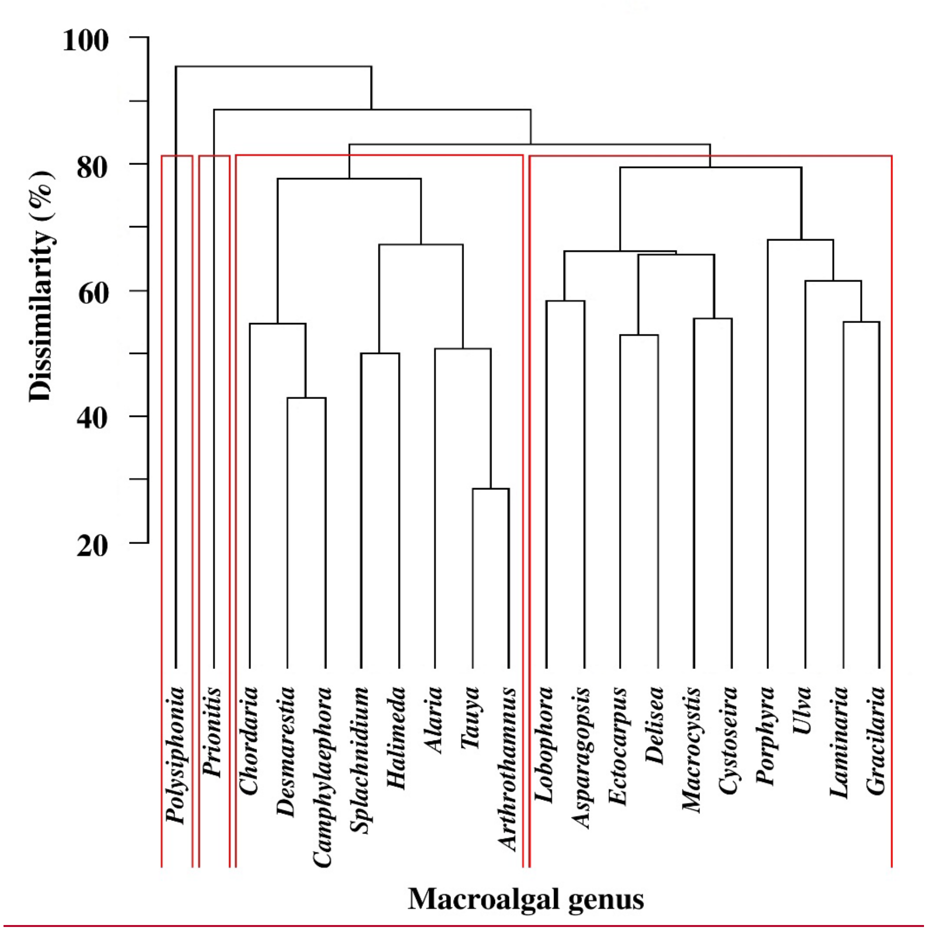

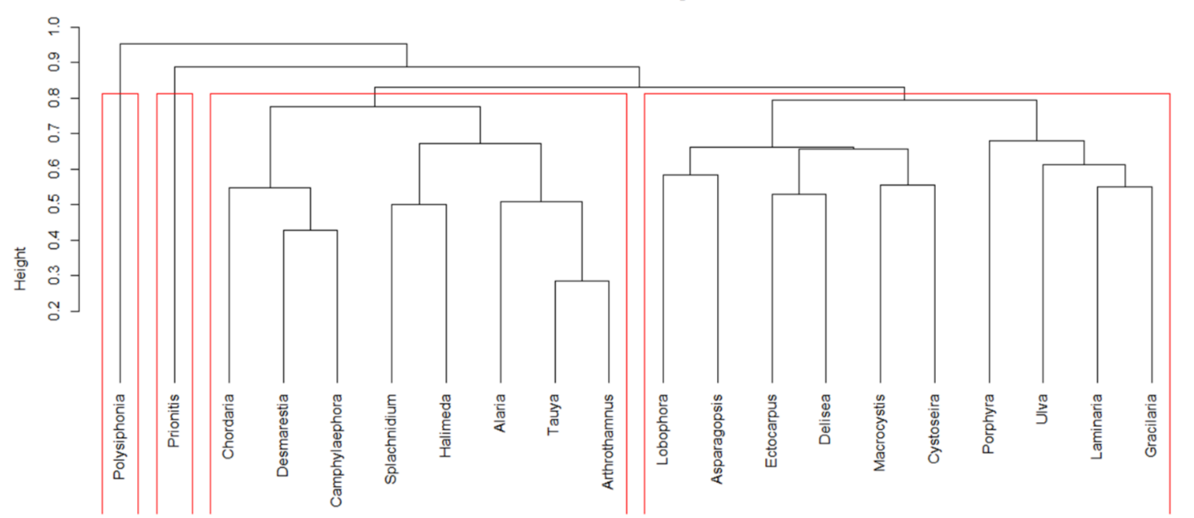
Height
0.2
0.3
0.4
0.5
0.6
0.7
0.8
0.9
1.0
Polysiphonia
Prionitis
Chordaria
Desmarestia
Camphylaephora
Splachnidium
Halimeda
Alaria
Tauya
Arthrothamnus
Lobophora
Asparagopsis
Ectocarpus
Delisea
Macrocystis
Cystoseira
Porphyra
Ulva
Laminaria
Gracilaria

1
