# Supplementary material for: A Functional Perspective Analysis of Macroalgae and Epiphytic Bacterial Community Interaction
Source: Front Microbiol. 2017 Dec 22;8:2561. doi: 10.3389/fmicb.2017.02561 (PMC5743738; doi:10.3389/fmicb.2017.02561)

SUPPLEMENTARY MATERIAL

Figure S3. Cluster analysis of the epiphytic bacteria, at the family level, associated to the ~~for the~~ different methodological approaches. The dissimilarity analysis is based on the Bray-Curtis index and a cut-off point of 7~~3~~0% dissimilarity was established (Table S5). ~~applied to establish differences between the three groups of methodologies.~~

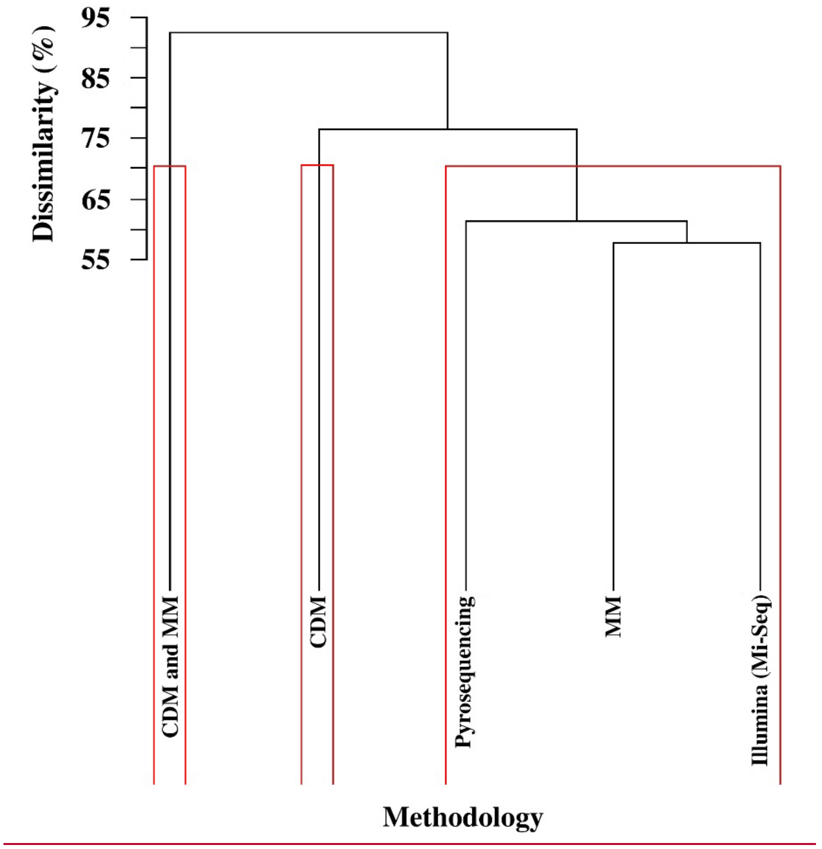

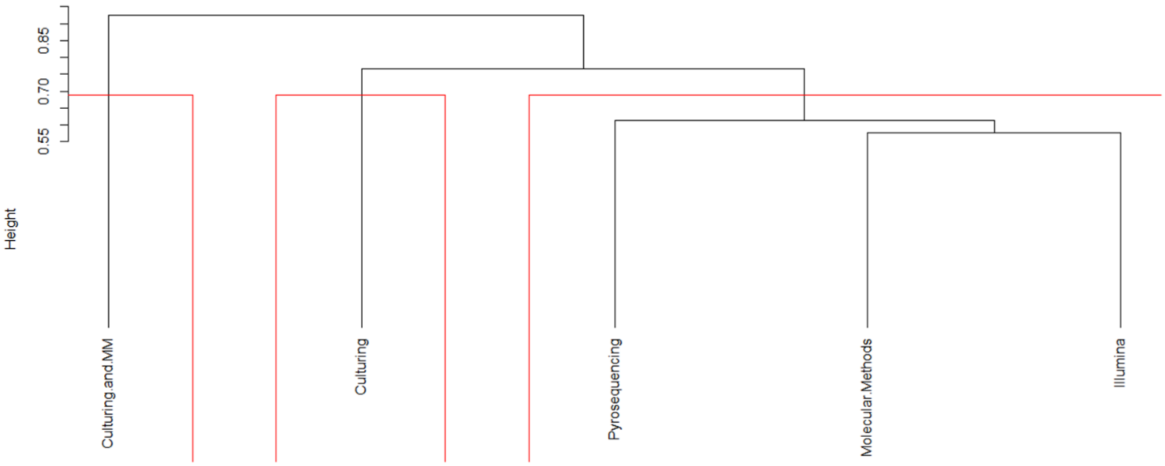

Supplement: Supplementary file 12 [file Image3.PDF]
